# Review of Canadian species of the genus *Dinaraea* Thomson, with descriptions of six new species (Coleoptera, Staphylinidae, Aleocharinae, Athetini)

**DOI:** 10.3897/zookeys.327.5908

**Published:** 2013-08-30

**Authors:** Jan Klimaszewski, Reginald P. Webster, David W. Langor, Caroline Bourdon, Jenna Jacobs

**Affiliations:** 1 Natural Resources Canada, Canadian Forest Service, Laurentian Forestry Centre, 1055 du P.E.P.S., P.O. Box 10380, Stn. Sainte-Foy, Québec, Quebec G1V 4C7, Canada; 2 24 Mill Stream Dr., Charters Settlement, New Brunswick E3C 1X1, Canada; 3 Natural Resources Canada, Canadian Forest Service, Northern Forestry Centre, 5320-122 Street, Edmonton, Alberta T6H 3S5, Canada; 4 Département des sciences biologiques, Université du Québec à Montréal, CP 8888, succursale Centre-ville, Montréal, Quebec H3C 3P8, Canada

**Keywords:** Canada, Coleoptera, Dinaraea, Staphylinidae, taxonomy, identification

## Abstract

Twelve species of the genus *Dinaraea* Thomson are recognized in the Nearctic region, ten of which occur in Canada, all east of the Rocky Mountains. Six species are herein described as new to science: *Dinaraea bicornis* Klimaszewski & Webster, **sp. n.**; *Dinaraea curtipenis* Klimaszewski & Webster, **sp. n.**; *Dinaraea longipenis* Klimaszewski & Webster, **sp. n.**; *Dinaraea quadricornis* Klimaszewski & Webster, **sp. n.**; *Dinaraea worki* Klimaszewski & Jacobs, **sp. n.**; and *Dinaraea piceana* Klimaszewski & Jacobs, **sp. n.** Four formerly described species are confirmed in Canada: *Dinaraea angustula* (Thomson), *Dinaraea backusensis* Klimaszewski & Brunke, *Dinaraea borealis* Lohse, and *Dinaraea pacei* Klimaszewki & Langor. The previously unknown male of *Dinaraea borealis* Lohse and female of *Dinaraea backusensis* are described. All species are illustrated with colour habitus images and black and white images of the median lobe of the aedeagus and spermatheca, and tergite VIII and sternite VIII of both sexes. New habitat and distribution data are presented and a key to all Nearctic species of the genus is provided.

## Introduction

*Dinaraea* Thomson is a small genus with mainly subcortical species distributed in the Palaearctic and Nearctic regions. There were nine valid species recorded from the Palaearctic region (Lohse 1974, Smetana 2004), and six species recorded from the Nearctic region prior to this publication. The Nearctic species, *Dinaraea nomensis* Casey, was previously transferred to the genus *Boreophilia* Benick (Lohse et al. 1990). The remaining six valid Nearctic species previously reported are: *Dinaraea planaris* (Mäklin) from Alaska (Mäklin 1852), *Dinaraea subdepressa* (Bernhauer) from New Hampshire (Bernhauer 1907), *Dinaraea borealis* Lohse described from Quebec (Lohse et al. 1990), *Dinaraea pacei* Klimaszewski & Langor from Newfoundland (Klimaszewski et al. 2011), *Dinaraea backusensis* Klimaszewski & Brunke from Ontario (Brunke et al. 2012), and the adventive Palaearctic species *Dinaraea angustula* (Gyllenhal) (Muona 1984, Klimaszewski et al. 2010, Brunke et al. 2012). We were not able to examine *Dinaraea planaris* (Mäklin) but instead used the redescription and drawing of the median lobe of the aedeagus of this species provided by Lohse and Smetana (1985). During the course of the present study we discovered six additional species new to science from Canada, all distributed east of the Rocky Mountains. Many species of this genus may have implications for forest pest management as potential natural enemies of bark beetles and other economically important subcortical insects.

## Material and methods

We examined about 250 specimens of *Dinaraea* in this study. Nearly all specimens were dissected. Genitalic structures, tergite VIII, and sternite VIII were dehydrated in absolute alcohol, mounted in Canada balsam on celluloid microslides, and pinned with the specimens from which they originated. Specimens and dissected structures were examined using a Nikon SMZ 1000 stereomicroscope. Photographs were taken using an image processing system (Nikon SMZ 1500 stereoscopic microscope, Nikon Digital Camera DXM 1200F, and Adobe Photoshop software). Habitus photographs of all included species are provided, while genitalia are illustrated only for those species whose genitalia have not been shown in recent publications. In the species accounts, distributions are given by province or state (Canada, U.S.A.) or by country (elsewhere). These territories are abbreviated using Canada Post and United States Postal Service standards. Data for distribution maps were extracted from specimens in collections, as well as from literature records. Geographic coordinates were standardized using NAD83 datum, and maps were projected onto a Lambert Conic Conformal using ESRI ArcMap version 10 for Windows.

Morphological terminology mainly follows that used by Seevers (1978) and Ashe (2000). The ventral (= parameral) part of the median lobe of the aedeagus is considered to be the part of the bulbus containing the foramen mediale, the entrance of the ductus ejaculatorius, and the adjacent venter of the tubus; the opposite side is referred to as the dorsal (= abparameral) part.

Specimens were examined from the following collections:

AAFC-SJAgriculture and Agri-Food Canada, St. John’s, Newfoundland and Labrador, Canada

ACPEAgriculture and Agri-Food Canada, Charlottetown, Prince Edward Island, Canada

CNCCanadian National Collection of Insects, Ottawa, Ontario, Canada

DEBUGuelph University Collection, Guelph University, Guelph, Ontario, Canada (temporarily at LFC)

FMNHField Museum of Natural History, Chicago, Illinois, USA

LFCLaurentian Forestry Centre, Canadian Forest Service, Québec, Quebec, Canada

MUNMemorial University of Newfoundland, St. John’s, Newfoundland and Labrador, Canada (currently on long-term loan to David Langor, Canadian Forest Service, Edmonton, Alberta, Canada)

NBMNew Brunswick Museum, Saint John, New Brunswick, Canada

NOFCNorthern Forestry Centre, Canadian Forest Service, Edmonton, Alberta, Canada

NSMNova Scotia Museum, Halifax, Nova Scotia, Canada

RWCReginald Webster Collection, 24 Mill Stream Dr., Charters Settlement, New Brunswick, Canada

UAMMuseum of the North, University of Alaska, Fairbanks, Alaska, USA

## Taxonomic review

### Genus Dinaraea

Thomson, 1858: 33

http://species-id.net/wiki/Dinaraea

#### Type species.

*Homalota aequata* Erichson, 1837.

#### Diagnosis.

Body subparallel, flattened, integument with distinct meshed microsculpture, punctation distinct; head large, subquadrate to slightly elongate, genae usually longer than eyes, suborbital carina absent; clypeus long, nearly horizontal, not depressed as in other genera of Athetini; mandibles strong and slightly curved apically, left one with smooth edge and right one with small subapical tooth in apical third on inner edge; maxillary palpus 4-segmented, basal segment small and apical segment short and needle-shaped; lacinia and galea moderately elongate, lacinia with fringe of long hairs subapically; labial palpus 3-segmented; ligula V-shaped and each arm narrow; pronotum usually of trapezoidal form, usually widest in apical third, with pubescence at midline of disc directed posteriad in apical two-thirds, anteriad in basal third, and laterad at sides; elytra short, at suture as long as pronotum or only slightly longer; abdominal tergites 3-5 each with distinct basal impression; tarsal formula 4-5-5; male tergite VIII in majority of species with small teeth at apical margin and sometimes also in adjacent area of disc; median lobe of aedeagus with athetine bridge and of a simple form, with inconspicuous structures of internal sac; spermatheca with conical capsule and long slim stem coiled posteriorly.

#### Comments.

Based on the morphology of the male tergite VIII, two groups of species are recognized. One group, consisting of the majority of species, is characterized by tergite VIII bearing two-to-several small teeth on the apical margin. Species in the second group (*Dinaraea borealis*, *Dinaraea curtipenis*, *Dinaraea longipenis*, *Dinaraea planaris*, *Dinaraea subdepressa*), have tergite VIII truncate and without teeth on the apical margin.

#### Biology.

Many species are known from the subcortical galleries of other insects but their diet is unknown.

### Checklist of Nearctic species

1. *Dinaraea angustula* (Gyllenhal), adventive in Nearctic region [AB, LB, NB, NF, NS, ON, PE, QC, YT]

2. *Dinaraea backusensis* Klimaszewski & Brunke, in Brunke et al. 2012: 175 [MA, NB, ON]

3. *Dinaraea bicornis* Klimaszewski & Webster, sp. n. [NB, ON]

4. *Dinaraea borealis* Lohse et al., 1990: 198 [NB, ON, QC]

5. *Dinaraea curtipenis* Klimaszewski & Webster, sp. n. [NB]

6. *Dinaraea longipenis* Klimaszewski & Webster, sp. n. [NB]

7. *Dinaraea pacei* Klimaszewski & Langor, in Klimaszewski et al. 2011: 159 [AB, BC, LB, NB, QC]

8. *Dinaraea piceana* Klimaszewski & Jacobs, sp. n. [QC]

9. *Dinaraea planaris* (Mäklin 1852: 309) [AK, YT]

10. *Dinaraea subdepressa* (Bernhauer 1907: 386) [NH]

11. *Dinaraea quadricornis* Klimaszewski & Webster, sp. n. [NB]

12. *Dinaraea worki* Klimaszewski & Jacobs, sp. n. [QC]

### Key to Nearctic species

(Excluding *Dinaraea planaris* (Mäklin), which is only known from the lectotype that was not available for study; see illustration of median lobe of the aedeagus (Fig. 11b (original image) and Fig. 11a (a redescription in Lohse and Smetana (1985))). This species sensu Lohse and Smetana (1985) keys to couplet with *Dinaraea pacei* but has a different shape of the median lobe of aedeagus).

**Table:** 

1	Elytra at suture shorter than pronotum (Figs 1a, 4a, 10a)	2
–	Elytra at suture at least as long as pronotum (e.g., Figs 2a, 3a)	4
2	Maximum width of elytra equal to maximum width of pronotum (Fig. 4a); median lobe of aedeagus and spermatheca as illustrated (Fig. 4b, e)	*Dinaraea worki* Klimaszewski & Jacobs, sp. n.
–	Maximum width of elytra greater than maximum width of pronotum (Figs 1a, 10a)	3
3	Pronotum trapezoidal in shape, broadest in apical third, punctures very dense, pronotum appearing matte (Fig. 1a); male tergite VIII with two teeth (Fig. 1c); median lobe of aedeagus and spermatheca as illustrated (Fig. 1b, e)	*Dinaraea bicornis* Klimaszewski & Webster, sp. n.
–	Pronotum rectangular in shape, broadest at middle (Fig. 10a); male tergite VIII without teeth (Fig. 10c); median lobe of aedeagus as illustrated (Fig. 10b); female undescribed	*Dinaraea longipenis* Klimaszewski & Webster, sp. n.
4	Antennal articles VIII-X strongly transverse (Figs 2a, 3a, 8a, 9a)	5
–	Antennal articles VIII-X moderately transverse (Figs 5a, 6a, 7a)	8
5	Pronotum rectangular in shape, broadest at middle, punctures very dense (pronotum appearing matte) and distanced from each other by a diameter of a puncture (Fig. 2a); male tergite VIII, median lobe of aedeagus, and spermatheca as illustrated (Fig. 2c, b, e)	*Dinaraea quadricornis* Klimaszewski & Webster, sp. n.
–	Pronotum trapezoidal in shape, broadest in apical third, punctures moderately dense, distanced from each other by more than diameter of a puncture (Figs 3a, 8a, 9a, 10a); genital structures different (e.g., Figs 3b, c, h; 9b, e; 10b)	6
6	Body moderately broad (Fig. 3a); male tergite VIII with four apical teeth (Fig. 3d); tubus of median lobe of aedeagus broad and rounded apically (Fig. 3b, h); spermatheca as illustrated (Fig. 3c)	*Dinaraea backusensis* Klimaszewski & Brunke
-	Tubus of median lobe of aedeagus narrow and sharply pointed apically (Figs 8b, 9b); spermatheca as illustrated (Fig. 5e, 6e, 7e, 8e); females of *Dinaraea curtipenis* unknown	7
7	Body dark brown almost black (Fig. 9a); tubus of median lobe of aedeagus short and sinuate ventrally, and with broad sclerites of internal sac (Fig. 9b); female unknown	*Dinaraea curtipenis* Klimaszewski & Webster, sp. n.
–	Body dark brown with reddish tinge (Fig. 8a); tubus of median lobe of aedeagus long and straight ventrally, and with narrow sclerites of internal sac (Fig. 8b); spermatheca as illustrated (Fig. 8e)	*Dinaraea borealis* Lohse
8	Pronotum trapezoidal in shape, broadest in apical third (Figs 5a, 7a); male tergite VIII with or without apical teeth, but never with additional teeth-like structures in subapical part of disc (Figs 5c, 7c); spermatheca as illustrated (Figs 5e, 7e)	9
–	Pronotum rectangular in shape, broadest at middle (Fig. 6a); male tergite VIII with four apical teeth and additional teeth-like structures in subapical part of disc (Fig. 6c); spermatheca as illustrated (Fig. 6e)	*Dinaraea angustula* (Gyllenhal)
9	Pronotum dark brown and elytra yellowish-brown (Fig. 5a); male tergite VIII, median lobe of aedeagus and spermatheca as illustrated (Fig. 5b, c, e)	*Dinaraea piceana* Klimaszewski & Jacobs, sp. n.
–	Pronotum and elytra dark brown (Fig. 7a); genital structures differently shaped	10
10	Median lobe of aedeagus with tubus slightly sinuate apically and slightly produced ventrally at apex, internal sac structures short and straight (Fig. 13a); male tergite VIII without apical teeth (Fig. 13b), sternite VIII as illustrated (Fig. 13c)	*Dinaraea subdepressa* (Bernhauer)
–	Median lobe of aedeagus with tubus sinuate and strongly produced ventrally, internal sac structures long and arcuate (Fig. 7b); male tergite VIII with small apical teeth (Fig. 7c); spermatheca S-shaped (Fig. 7e)	*Dinaraea pacei* Klimaszewski & Langor[based on comparison of *Dinaraea pacei* with the Yukon specimen (CNC), which was compared with the lectotype of *Dinaraea planaris* by A. Lohse and Smetana (1985), there are no major external differences between the two species and they key to the same couplet. The two species differ by the shape of median lobe of aedeagus. See comments under *Dinaraea pacei*]

### 1. Dinaraea bicornis

Klimaszewski & Webster sp. n.

http://zoobank.org/299D97AC-B15D-4D4A-A4DD-F11E2E8DA77A

http://species-id.net/wiki/Dinaraea_bicornis

Fig. 1a–g , Map 1

#### HOLOTYPE

(male): **CANADA**, **NEW BRUNSWICK**, York Co., Kingsclear, Mazerolle Settlement, 45.872288°N, 66.83105°W, 9.IV.2006, R.P. Webster // Margin of stream, in litter at base of northern white cedar (LFC). **PARATYPES**: **CANADA**, **NEW BRUNSWICK**: Charlotte Co., 5 km NW of Pomeroy Ridge, 45.3059°N, 67.4343°W, 5.VI.2008, R.P. Webster // Red maple and eastern white cedar swamp, in moss & leaf litter near small vernal pools (RWC) 1 male; Charlotte Co., 3.5 km NW of Pomeroy Ridge, 45.3087°N, 67.4362°W, 16.VI.2008, R.P. Webster // Red maple swamp, in leaves and moss near small vernal pools (RWC) 1 female; York Co., Canterbury, Trail to Browns Mtn. Fen., 45.9033°N, 67.6260°W, 2.V.2005 // Mixed forest with cedar, margin of vernal pond in moist leaf litter (LFC) 1 sex undetermined; York Co., New Maryland, off Hwy 2, E of Baker Brook, 45.8760°N, 66.6252°W, 26.IV.2005, R.P. Webster // Old growth cedar swamp, in moss and litter at base of cedar (RWC) 1 female; York Co., NE of Exit 271 off Hwy 2, 45.8776°N, 66.8254°W, 8.VI.2008, R.P. Webster // Alder swamp with poplar, sifting leaf litter & moss near vernal pool (RWC) 1 female; **ONTARIO**: Leeds and Grenville. Co., 2 km SE Spencerville, 30.IV.1979, A. & Z. Smetana (CNC) 1 male, 1 female.

#### Etymology.

The specific name *bicornis* means ‘with two horns’ in allusion to the two teeth on the male tergite VIII.

#### Diagnosis.

**Figure 1.**
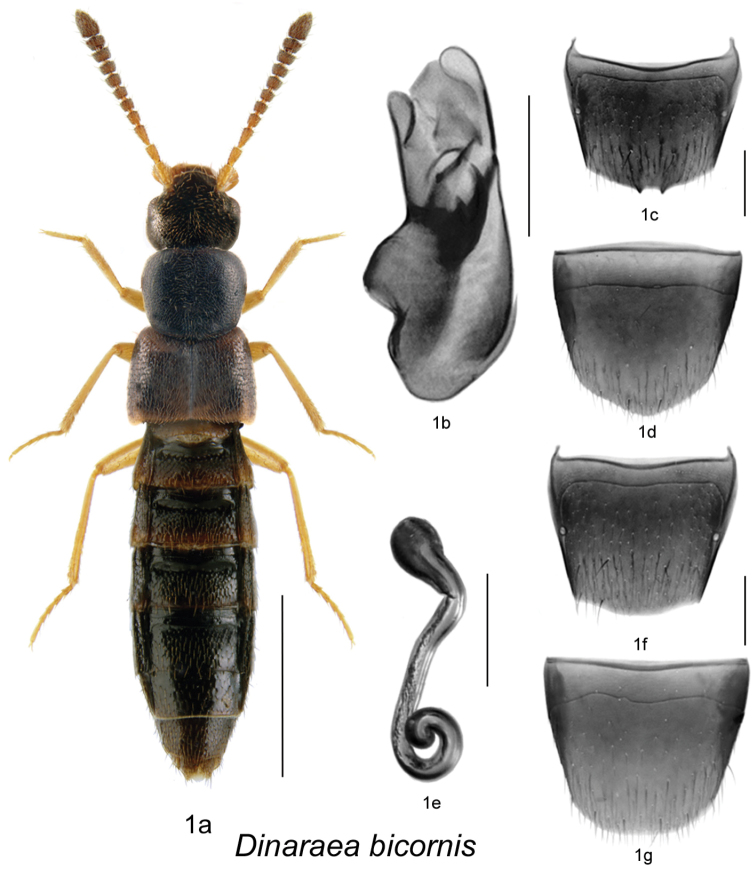
*Dinaraea bicornis* Klimaszewski & Webster, sp. n.: **a** habitus **b** median lobe of aedeagus in lateral view **c** male tergite VIII **d** male sternite VIII **e** spermatheca in lateral view **f** female tergite VIII **g** female sternite VIII. Habitus scale bar = 1.0 mm, other scale bars = 0.2 mm.

*Dinaraea bicornis* (habitus Fig. 1a) may be distinguished from congeners by the following combination of characters: body length 3.1-3.4 mm; head and pronotum matte with dense microsculpture; pronotum broadest at apical third; elytra at suture shorter than pronotum, with asperate punctation; antennal articles 7-10 strongly transverse; male tergite VIII with two small sharp apical teeth (Fig. 1c); median lobe of aedeagus with straight and short tubus narrowly rounded apically (Fig. 1b); spermatheca with pear-shaped capsule and moderately deep apical invagination, stem narrow, long and looped posteriorly, slightly swollen at apex (Fig. 1e).

#### Description.

Body length 3.1–3.4 mm; body dark brown with at least posterior part of elytra paler, with legs, antennae and labial palpi yellowish-brown; head and pronotum with dense microsculpture and appearing matte; elytral and particularly pronotal microsculpture less dense and their integument appears glossy; head about as broad as pronotum, genae slightly longer than eyes in dorsal view; pronotum broadest in apical third, slightly transverse, longer than elytra at suture; elytra transverse, shorter than pronotum at suture, truncate posteriorly; abdomen arcuate laterally, broadest in apical third; male tergite VIII with two small sharp medial teeth at apical margin (Fig. 1c), sternite VIII slightly produced posteriorly, antecostal suture arcuate (Fig. 1d); median lobe of aedeagus with short and straight venter of tubus and narrowly rounded apex (Fig. 1b); female tergite VIII slightly sinuate apically on each side of disc (Fig. 1f), sternite VIII rounded apically, antecostal suture sinuate (Fig. 1g); spermatheca with pear-shaped capsule and moderately deep apical invagination, stem narrow, long and looped posteriorly, slightly swollen at apex (Fig. 1e).

#### Distribution.

Known from New Brunswick and Ontario.

**Map 1–4.**
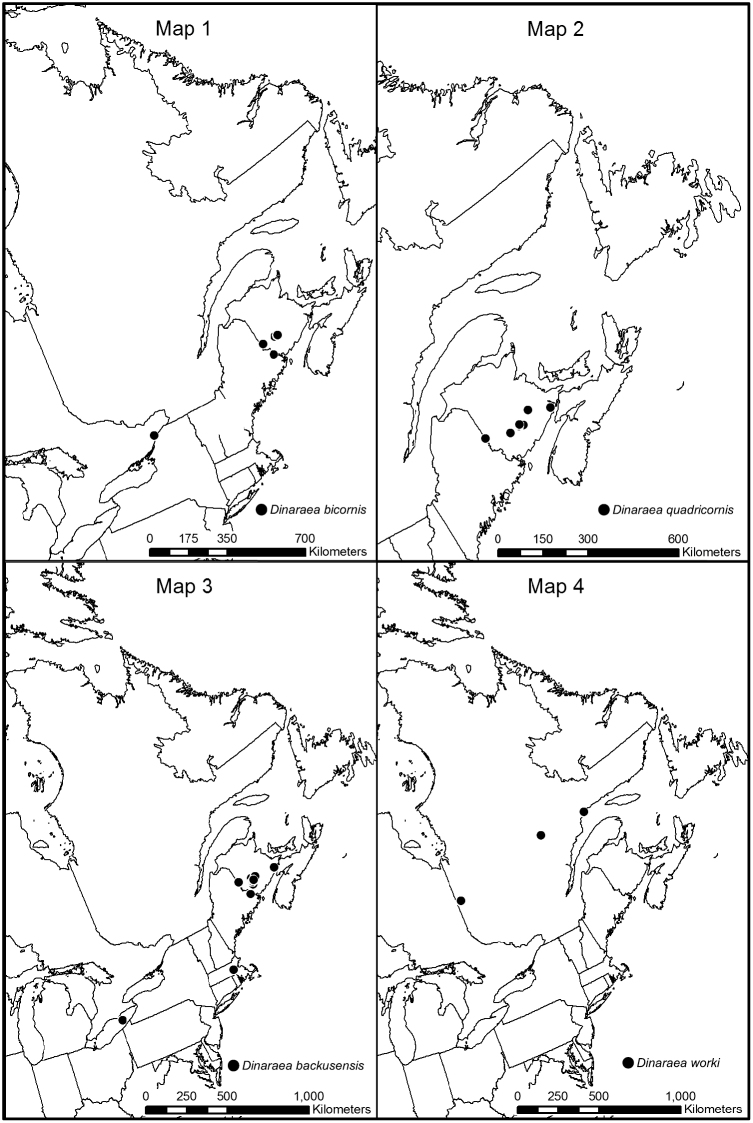
Distribution map of: **1** *Dinaraea bicornis* sp. n. **2** *Dinaraea quadricornis* sp. n. **3** *Dinaraea backusensis* **4** *Dinaraea worki* sp. n.

#### Collection and habitat data.

Most adults were collected in April and May, and one specimen in October. The New Brunswick specimens were found in moss and leaf litter near the edges of small vernal pools in forests containing eastern white cedar (*Thuja occidentalis* L.) and red maple (*Acer rubrum* L.), and sometimes alder and poplar.

### 2. Dinaraea quadricornis

Klimaszewski & Webster sp. n.

http://zoobank.org/F1252FB1-064A-49FF-8619-1C741088FED7

http://species-id.net/wiki/Dinaraea_quadricornis

Fig. 2a–g , Map 2

#### HOLOTYPE

(male): **CANADA**, **NEW BRUNSWICK**, Queens Co., Cambridge, W of Jemseg at “Trout Creek”, 45.8227°N, 66.1240°W, 3.VI.2007, R.P. Webster // Silver maple forest, under tight bark of *Ulmus americana* L. (White elm) (LFC) 1 male. **PARATYPES**: **CANADA**, **NEW BRUNSWICK**: Albert Co., Caledonia Gorge P.N.A., 45.7930°N, 64.7764°W, 1.VII.2011, R.P. Webster // Small rocky clear-cold river (Caledonia Creek), sifting drift material (tree bud material) in eddy area (RWC) 1 male; Albert Co., Caledonia Gorge P.N.A., 45.7941°N, 64.7736°W, 13.IX.2011, R.P. Webster // Near Crooked Creek, mixed forest (red spruce & yellow birch) in decaying mushrooms (NBM) 1 male; Carleton Co., Richmond, Hovey Hill Protected Area, 46.1115°N, 67.7770°W, 24.V.2005, R.P. Webster // Hardwood forest, under bark of beech log (RWC) 1 male; Queens Co., Canning, Grand Lake near Scotchtown, 45.8762°N, 66.1816°W, 30.IV.2006, R.P. Webster // Oak forest, under bark of oak (RWC) 1 female (NBM) 1 female; same locality and habitat data, and collector except 25.V.2006 (RWC) 1 male; Queens Co., Cranberry Lake P.N.A., 46.1125°N, 65.6075°W, 5–11.VI.2009 // R. Webster & M.-A. Giguère, Red oak forest, Lindgren funnel trap (RWC) 1 female; same locality and habitat data, and collectors except 25.VI-1.VII.2009 (RWC) 1 male; Queens Co. Grand Lake Meadows P.N.A., 45.8227°N, 66.1209°W, 17–30.VIII.2011, C. Hughes & R.P. Webster // Old silver maple forest and seasonally flooded marsh, Lindgren funnel trap (NBM) 1 female; Sunbury Co., Lakeville Corner, 45.9007°N, 66.2423°W, 10.IX.2006, R.P. Webster // Silver maple forest, on ridge with oaks, on gilled mushroom (RWC) 1 male; Sunbury Co., Gilbert Island, 45.8769°N, 66.2954°W, 18.VII.2012, C. Hughes & R.P. Webster // Hardwood forest on island, under bark of hardwood (AFC) 1 female York Co., New Maryland, Charters Settlement, 45.8340°N, 66.7450°W, 29.III.2006, R.P. Webster (LFC) 1 female; same locality data except 22.IV.2006, R.P. Webster // Mixed forest in wood pile under bark of spruce (RWC) 1 male; same locality data except 30.IV.2006, R.P. Webster // Mixed forest in wood pile under bark of spruce (RWC) 1 male, 1 female; York Co., New Maryland, Charters Settlement, 45.8331°N, 66.7410°W, 14.IV.2006, R.P. Webster // Mixed forest, under bark of spruce (NBM) 1 female; York Co., New Maryland, Charters Settlement, 45.8404°N, 66.7360°W, 27.V.2008, R.P. Webster // Mixed forest, under bark of spruce (RWC) 1 female.

#### Etymology.

The specific name *quadricornis*, means ‘with four horns’ in allusion to the four teeth on the male tergite VIII.

#### Diagnosis.

**Figure 2.**
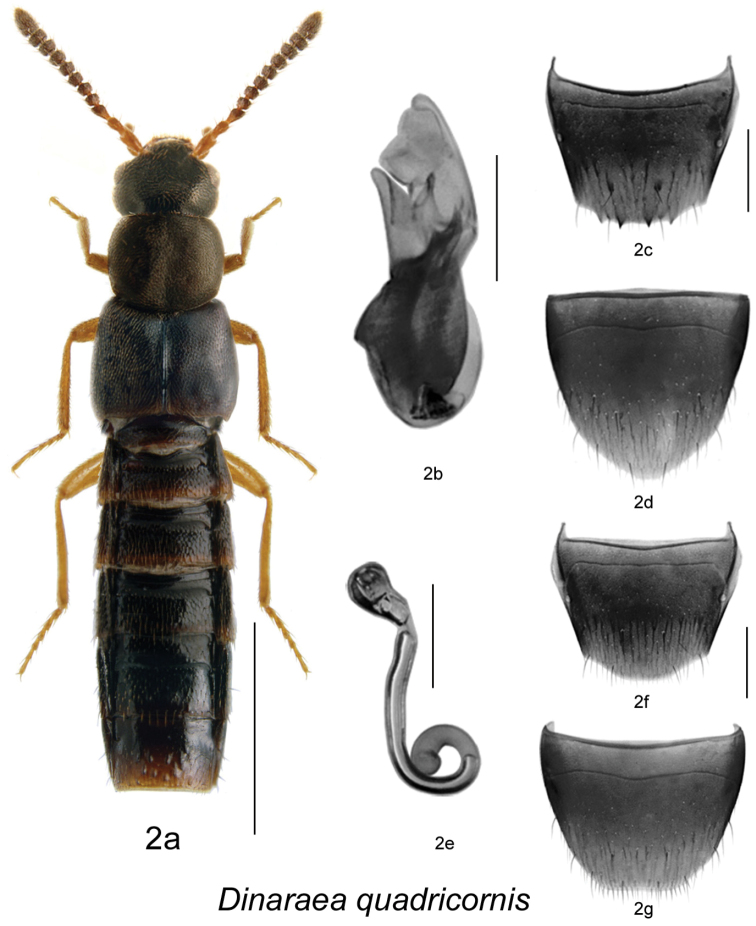
*Dinaraea quadricornis* Klimaszewski & Webster, sp. n.: **a** habitus **b** median lobe of aedeagus in lateral view **c** male tergite VIII **d** male sternite VIII **e** spermatheca in lateral view **f** female tergite VIII **g** female sternite VIII. Habitus scale bar = 1.0 mm, other scale bars = 0.2 mm.

*Dinaraea quadricornis* (habitus Fig. 2a) may be distinguished from congeners by the following combination of characters: body length 3.2–3.5 mm; head, pronotum and elytra matte with dense microsculpture; pronotum broadest at middle; elytra at suture as long as pronotum, with dense punctation similar to that on pronotum; antennal articles 7–10 strongly transverse; male tergite VIII with four small sharp apical teeth (Fig. 2c); median lobe of aedeagus with straight and short tubus, narrowly rounded apically and slightly produced ventrally (Fig. 2b); spermatheca with pear-shaped capsule and moderately deep apical invagination, stem narrow, long and looped posteriorly, slightly swollen at apex (Fig. 2e).

#### Description.

Body length 3.2–3.5 mm; body dark brown with legs, antennae (at least basally) and labial palpi yellowish- or reddish-brown; head, pronotum and elytra matte, elytra less so, with dense microsculpture; abdominal microsculpture moderately dense and integument glossy; head about as broad as pronotum, genae slightly longer than eyes in dorsal view; pronotum broadest at middle, slightly transverse, as long as elytra at suture; elytra transverse, truncate posteriorly; abdomen arcuate laterally, broadest at middle; male tergite VIII with four small sharp teeth at apical margin (Fig. 2c), sternite VIII slightly produced posteriorly, antecostal suture sinuate (Fig. 2d); median lobe of aedeagus with short and straight tubus, venter of tubus and narrowly rounded apex slightly produced ventrally (Fig. 2b); female tergite VIII slightly sinuate apically on each side of the disc (Fig. 2f), sternite VIII truncate apically, antecostal suture sinuate (Fig. 2g); spermatheca with pear-shaped capsule and moderately deep apical invagination, stem narrow, long and looped posteriorly, slightly swollen at apex (Fig. 2e).

#### Distribution.

Known only from New Brunswick.

#### Collection and habitat data.

Adults were collected from March to September in several microhabitats: under tight bark of white elm in a silver maple (*Acer saccharinum* L.) forest; in a gilled mushroom located on a ridge with oaks in a silver maple forest; in a hardwood forest with silver maple and butternut (*Juglans cinerea* L.); under the bark of a hardwood tree; in a wood pile; under the bark of spruce (*Picea* sp.) in a mixed forest; under the bark of a spruce log in an old mixed forest; in a hardwood forest under the bark of a beech (*Fagus grandifolia* Ehrh.) log; in decaying mushrooms in a mixed forest with red spruce (*Picea rubens* Sarg.) and yellow birch (*Betula alleghaniensis* Britt.); and in a red oak (*Quercus rubra* L.) forest under the bark of red oak. Flying adults were also captured in Lindgren funnel traps in an old red oak forest and in an old silver maple forest and seasonally flooded marsh. One individual was sifted from drift material (tree buds) in an eddy area along a fast-flowing, clear, cold and rocky river.

### 3. Dinaraea backusensis

Klimaszewski & Brunke

http://species-id.net/wiki/Dinaraea_backusensis\according to Klimaszewski et al 2013

Fig. 3a–g , Map 3

Dinaraea backusensis Klimaszewski & Brunke, in Brunke et al. 2012: 175.

#### HOLOTYPE

(male): **CANADA**, **ONTARIO**, Haldimand-Norfolk Reg., 6 km W of Saint Williams, Backus Woods, Wetland trail, sugar maple-dominated mesic forest, 42.3954°N, 80.2934°W, 2.IV.2010, A. Brunke // Accession No. 00331025 (DEBU). Holotype examined.

**Figure 3.**
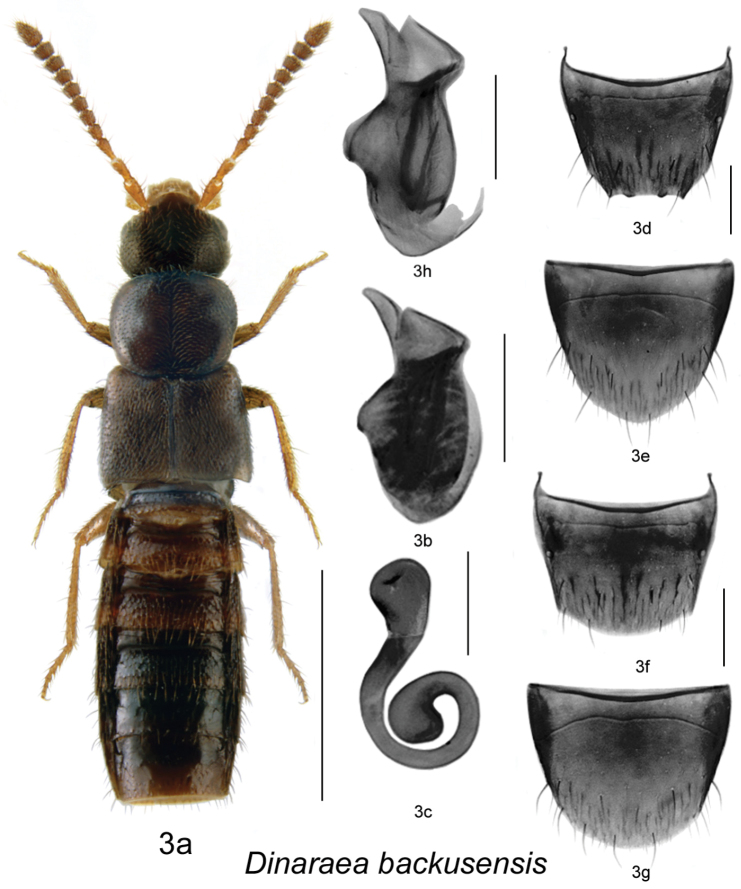
*Dinaraea backusensis* Klimaszewski & Brunke: **a** habitus **b** median lobe of aedeagus in lateral view **c** male tergite VIII **d** male sternite VIII **e** spermatheca in lateral view **f** female tergite VIII **g** female sternite VIII **h** median lobe of aedeagus in lateral view based on holotype. Habitus scale bar = 1.0 mm, other scale bars = 0.2 mm.

**Diagnosis.** *Dinaraea backusensis* (habitus Fig. 3a) may be distinguished from congeners by the following combination of characters: body length 2.4–2.6 mm; head, pronotum and elytra moderately glossy with dense microsculpture; pronotum broadest in about apical third and narrowest at base; elytra at suture as long as pronotum, with dense punctation similar to that on pronotum; antennal articles 7–10 moderately transverse; male tergite VIII with four small apical median teeth, median ones rounded (Fig. 3d); median lobe of aedeagus with straight and short tubus, narrowly rounded apically (Fig. 3b, h); spermatheca with large pear-shaped capsule and moderately shallow apical invagination, stem moderately long and looped posteriorly, apical end strongly swollen (Fig. 3c).

#### Description.

Body length 2.4–2.6 mm; body dark brown with legs, antennae (at least basally) and labial palpi yellowish- or reddish-brown; head, pronotum and elytra slightly glossy, with dense microsculpture; abdominal microsculpture moderately dense and integument glossy; head about as broad as pronotum, genae slightly longer than eyes in dorsal view; pronotum broadest in apical third, slightly transverse, as long as elytra at suture; elytra transverse, truncate posteriorly; abdomen arcuate laterally, broadest in middle; male tergite VIII with four small and short teeth at apical margin, all about the same size, median teeth rounded (Fig. 3d), sternite VIII slightly rounded posteriorly, antecostal suture arcuate and anterior margin concave (Fig. 3e); median lobe of aedeagus with short and straight venter of tubus and narrowly rounded apex (Fig. 3b, h); female tergite VIII broadly arcuate apically (Fig. 3f), sternite VIII rounded apically, antecostal suture arcuate (Fig. 3g); spermatheca with large pear-shaped capsule and moderately shallow apical invagination, stem moderately long, and looped posteriorly, apical part strongly swollen (Fig. 3c).

#### Distribution.

Known from Ontario and New Brunswick in Canada and Massachusetts in the USA.

#### Collection and habitat data.

Adults were collected from March to August mostly from under the bark of rotting logs (spruce, maples) in a variety of hardwood and mixed hardwood-conifer forest types. Some adults were found in polypore fungi on a large fallen basswood (*Tilia americana* L.) and in moss and litter at the base of cedar in an old-growth eastern white cedar swamp. Flying adults were also captured in Lindgren funnel traps in a red spruce forest with red maple and balsam fir (*Abies balsamea* (L.) Mill.) and in an old red pine (*Pinus resinosa* Ait.) forest.

#### Material examined.

**CANADA**: **NEW BRUNSWICK**: Albert Co., Caledonia Gorge P.N.A., 45.8175°N, 64.7770°W, 6.VII.2011, R.P. Webster // Mature hardwood forest, rotten sugar maple log, under bark (RWC) 1 male; Caledonia Gorge P.N.A., 45.7760°N, 64.7935°W, 1.VII.2011, R.P. Webster // Old-growth sugar maple & yellow birch forest, under bark of sugar maple log (NBM) 1 male; Caledonia Gorge P.N.A., 45.8380°N, 64.8484°W, 3.VII.2011, R.P. Webster // Old-growth sugar maple & yellow birch forest, under bark of sugar maple log (NBM) 1 male; Carleton Co., Jackson Falls, “Bell Forest Preserve”, 46.2200°N, 67.7231°W, 13.VIII.2006, R.P. Webster // Hardwood forest, on slightly dried *Pleurotus* sp., on dead standing maple (RWC) 1 female; Carleton Co., Jackson Falls, “Bell Forest Nature Preserve”, 46.2199°N, 67.7231°W, 7.VI.2007, R.P. Webster // Rich Appalachian hardwood forest, in polypore fungi on large fallen basswood (RWC) 1 male; Charlotte Co., 5 km NW of Pomeroy Ridge, 45.3059°N, 67.4343°W, 5.VI.2008, R.P. Webster // Red maple and eastern white cedar swamp, under bark of red maple (RWC) 1 female; Sunbury Co., Acadia Research Forest, 46.0188°N, 66.7450°W, 17.VIII.2007, R.P. Webster // Road 16 Control, Mature red spruce & red maple forest, under bark of red maple (RWC) 1 female; Sunbury Co., Acadia Research Forest, 45.9866°N, 66.3841°W, 24–30.VI.2009, R. Webster & M.-A. Giguère // Red spruce forest with red maple & balsam fir, Lindgren funnel trap (RWC) 1 male; York Co., New Maryland, Charters Settlement, 45.8342°N, 66.7452°W, 23.IV.2004, R.P. Webster (LFC) 1 male; York Co., New Maryland, Charters Settlement, 45.8340°N, 66.7450°W, 27.IV.2005, R.P. Webster // Mixed forest, in wood pile, under bark of spruce (RWC, LFC) 2 males, 1 female; York Co., New Maryland, Charters Settlement, 45.8395°N, 66.7391°W, 1.V.2004, R.P. Webster // Mixed forest, under bark of conifer log (RWC) 1 female; York Co., New Maryland, 45.8395°N, 66.7391°W, 6.VI.2006, R.P. Webster // Mixed forest, in fungus covered log (punky wood) (RWC) 1 female; York Co., New Maryland, 45.8395°N, 66.7391°W, 18.VI.2008, R.P. Webster // Mixed forest, in rotten log (RWC) 1 female; York Co., New Maryland, off Hwy 2, E of Baker Brook, 45.8760°N, 66.6252°W, 6.IV.2005, R.P. Webster // Old growth cedar swamp, in moss & litter at base of cedar (RWC) 1 male; York Co., 15 km E of Tracy, off Rt. 645, 45.6848°N, 66.8821°W, 4–16.VI.2010, R. Webster & C. MacKay // Old red pine forest, Lindgren funnel trap (RWC) 1 male; York Co., 14 km WSW of Tracy, S of Rt. 645, 45.6741°N, 66.8661°W, 9.VI.2010, R.P. Webster // Old mixed forest, under bark of rotten red maple log (RWC) 1 male. **USA**: **MASSACHUSETTS**: Framingham, 10.III.1947, C.A. Frost (CNC) 1 female.

### 4. Dinaraea worki

Klimaszewski & Jacobs sp. n.

http://zoobank.org/88174B76-3509-4188-A887-CFA9759B6D71

http://species-id.net/wiki/Dinaraea_worki

Fig. 4a–g , Map 4

#### HOLOTYPE

(male): **CANADA**, **QUEBEC**, Villebois, *Picea mariana*, coll. J. Jacobs, 2008, DB-ID 2913 (LFC). **PARATYPES**: **CANADA**, **QUEBEC**: Villebois, *Picea mariana*, coll. J. Jacobs, 2008, DB-ID 305 (LFC) 1 female; same data except: DB-ID 490 (LFC) 1 female; DB-ID 2749 (LFC) 1 female; DB-ID 2758 (LFC) 1 female; DB-ID 2765 (LFC) 1 female; DB-ID 2768 (LFC) 1 female; DB-ID 2770 (LFC) 1 female; DB-ID 2778 (LFC) 1 female; DB-ID 2780 (LFC) 1 female; DB-ID 2918 (LFC) 1 male; DB-ID 2994 (LFC) 1 female; Quebec, Sept Iles, 2.XI.1985 (LFC) 5 males, 7 females. **NON-TYPES**: **CANADA**, **QUEBEC**: Lac-St-Jean, Compagnie forestière Arbec, 50°22'54"N, 70°33'29"W, 12.VIII–25.VIII.2009, Annelage 2009, C. Hébert, pessière à mousses (LFC) 1 female; Lac-St-Jean, Compagnie forestière Arbec, 50°22'06"N, 70°33'22"W, 17.VI–01.VII.2009, Annelage 2009, C. Hébert, pessière à mousses (LFC) 1 female [last two females have slightly damaged spermathecae].

#### Etymology.

Named for Dr. Timothy Work, Université du Québec à Montréal, Quebec, Canada, who provided many insect specimens for this study and who has advanced the ecological knowledge of epigaeic beetles in Canada.

#### Diagnosis.

**Figure 4.**
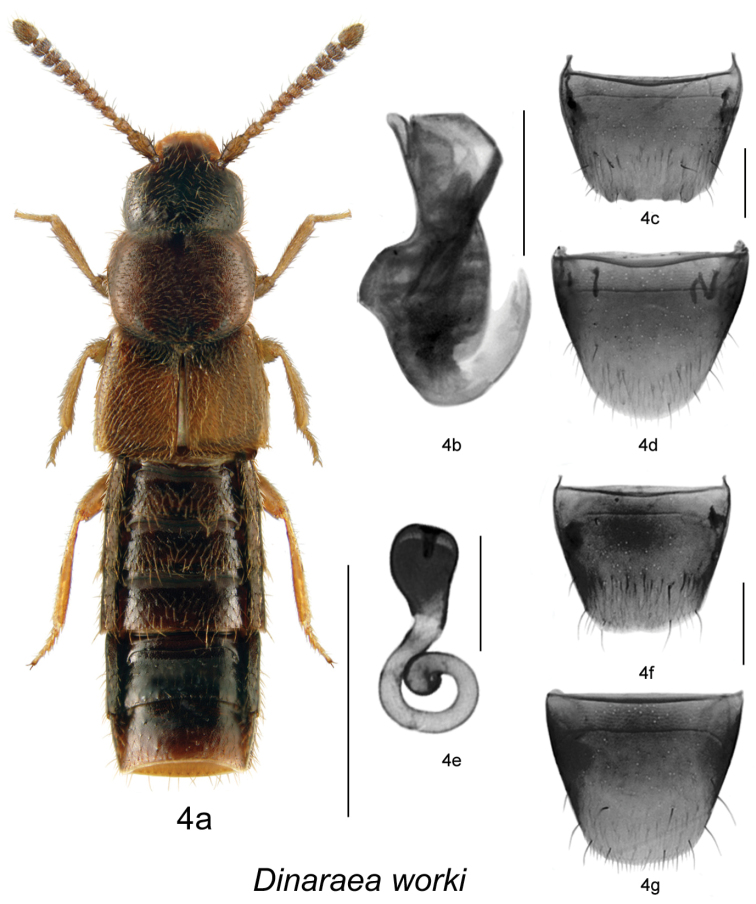
*Dinaraea worki* Klimaszewski & Jacobs, sp. n.: **a** habitus **b** median lobe of aedeagus in lateral view **c** male tergite VIII **d** male sternite VIII **e** spermatheca in lateral view **f** female tergite VIII **g** female sternite VIII. Habitus scale bar = 1.0 mm, other scale bars = 0.2 mm.

*Dinaraea worki* (habitus Fig. 4a) may be distinguished from congeners by the following combination of characters: body length 2.3–2.6 mm; head, pronotum and elytra moderately glossy with moderately dense microsculpture; pronotum broadest in middle and narrowest at base; elytra at suture as long as pronotum, with dense punctation similar to that on pronotum; antennal articles 7–10 strongly transverse; male tergite VIII with four small apical teeth, all short and rounded (Fig. 4c); median lobe of aedeagus with straight and short tubus narrowly rounded apically (Fig. 4b); spermatheca with large pear-shaped capsule and long apical invagination, stem short and looped posteriorly, with slightly swollen apical part (Fig. 4e).

#### Description.

Body length 2.3–2.6 mm; body dark brown with legs, antennae (at least basally), labial palpi and posterior part of elytra yellowish- or reddish-brown; head, pronotum and elytra moderately glossy, with moderately dense microsculpture; abdominal microsculpture moderately dense and integument more glossy; head about as broad as pronotum, genae slightly longer than eyes in dorsal view; pronotum broadest in middle, slightly transverse, about as long as elytra at suture; elytra transverse, truncate posteriorly; abdomen arcuate laterally, broadest in middle; male tergite VIII with four small rounded teeth at apical margin (Fig. 4c), sternite VIII rounded posteriorly, antecostal suture arcuate and anterior margin broadly arcuate (Fig. 4d); median lobe of aedeagus with short and straight venter of tubus and narrowly rounded apex (Fig. 4b); female tergite VIII slightly sinuate apically on each side of disc (Fig. 4f), sternite VIII rounded apically, antecostal suture sinuate (Fig. 4g); spermatheca with large pear-shaped capsule, and long apical invagination, stem short and looped posteriorly, with slightly swollen apical part (Fig. 4e).

#### Distribution.

Known from Quebec.

#### Collection and habitat data.

Several adults were collected in November. Specimens from western Quebec were collected from dead and decaying black spruce (*Picea mariana* Mill. (BSP)) in a boreal forest dominated by black spruce.

### 5. Dinaraea piceana

Klimaszewski & Jacobs sp. n.

http://zoobank.org/18BB3EFB-5A12-4A99-A6F6-31969051AEFE

http://species-id.net/wiki/Dinaraea_piceana

Fig. 5a–g , Map 7

#### HOLOTYPE

(male): **CANADA**, **QUEBEC**: Villebois, *Picea mariana*, coll. J. Jacobs, 2008, DB-ID 2797 (LFC) 1 male. **PARATYPES**: same data as the holotype except: DB-ID 333 (LFC) 1 female; DB-ID 847 (LFC) 1 male; DB-ID 1255 (LFC) 1 female; DB-ID 2722 (LFC) 1 female; DB-ID 1745 (LFC) 1 male; DB-ID 1747 (LFC) 1 female; DB-ID 2756 (LFC) 1 male; DB-ID 2774 (LFC) 1 female; DB-ID 2784 (LFC) 1 female; DB-ID 2785 (LFC) 1 male; DB-ID 2840 (LFC) 1 female; DB-ID 2844 (LFC) 1 female; DB-ID 2869 (LFC) 1 male; DB-ID 2870 (LFC) 1 male; DB-ID 2876 (LFC) 1 female; DB-ID 2879 (LFC) 1 male; DB-ID 2880 (LFC) 1 female; DB-ID 2884 (LFC) 1 male; DB-ID 2885 (LFC) 1 female; DB-ID 2893 (LFC) 1 female; DB-ID 2907 (LFC) 1 female; DB-ID 2908 (LFC) 1 male; DB-ID 2909 (LFC) 1 female; DB-ID 3265 (LFC) 1 male; MRC Manic, Réservoir Outardes 4, 50°37'N, 69°35'W, 2.VII–10.VII.2007, Chaire Côte-Nord, J.P. Légaré, Bloc Abitibi Sud, CP2 (CJSP) 3–10 m, Piège à impact, 2007-3-0520 (LFC) 1 female.

#### Etymology.

*Piceana* is an adjective derived from the tree name *Picea mariana* Mill. (BSP), in allusion to the black spruce forest where it was found.

#### Diagnosis.

**Figure 5.**
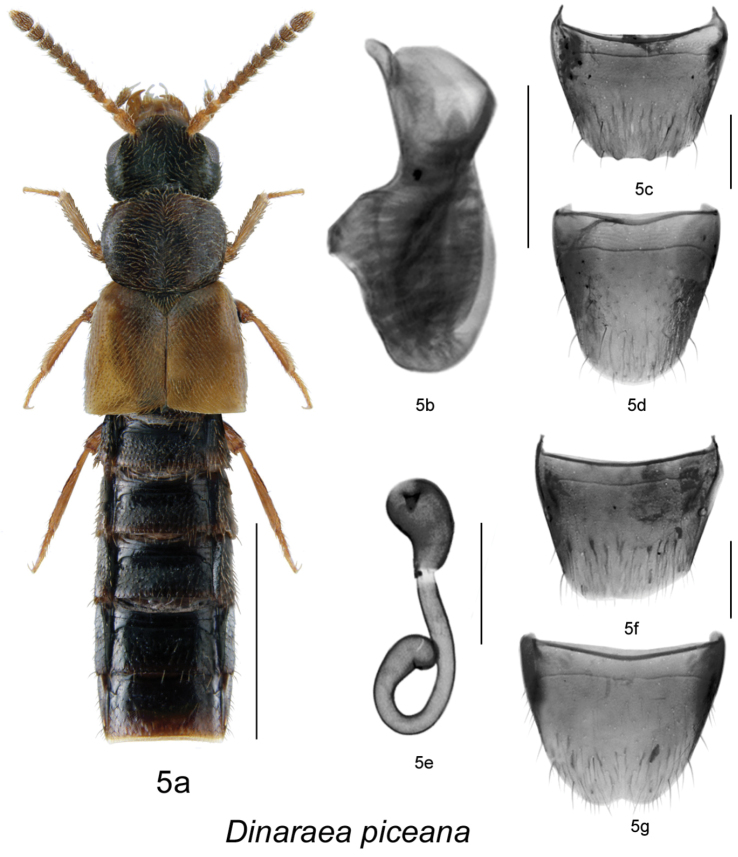
*Dinaraea piceana* Klimaszewski & Jacobs, sp. n.: **a** habitus **b** median lobe of aedeagus in lateral view **c** male tergite VIII **d** male sternite VIII **e** spermatheca in lateral view **f** female tergite VIII **g** female sternite VIII. Habitus scale bar = 1.0 mm, other scale bars = 0.2 mm.

*Dinaraea piceana* (habitus Fig. 5a) may be distinguished from congeners by the following combination of characters: body length 3.1–3.3 mm; head, pronotum and elytra matte with dense microsculpture; pronotum broadest in apical third and narrowest at base; elytra at suture slightly longer than pronotum, with dense punctation similar to that on pronotum; antennal articles 7–10 moderately transverse; male tergite VIII with four small apical teeth, all short and rounded (Fig. 5c); median lobe of aedeagus with straight and short tubus narrowly rounded apically and slightly produced ventrally (Fig. 5b); spermatheca with elongate pear-shaped capsule, and moderately long apical invagination, stem long, sinuate and looped posteriorly, with slightly larger apical part (Fig. 5e).

#### Description.

Body length 3.1–3.3 mm; body dark brown with legs, antennae (at least basally), labial palpi and elytra yellowish- or reddish-brown; head, pronotum and elytra matte, with dense microsculpture; abdominal microsculpture less dense than that of pronotum and elytra and integument more glossy; head about as broad and as large as pronotum, genae slightly longer than eyes in dorsal view; pronotum broadest in apical third, slightly transverse, longer than elytra at suture; elytra transverse, truncate posteriorly; abdomen subparallel; male tergite VIII with four small rounded teeth at apical margin (Fig. 5c), sternite VIII rounded posteriorly, antecostal suture and anterior margin of disc sinuate (Fig. 5d); median lobe of aedeagus with short and straight venter of tubus and narrowly rounded apex (Fig. 5b); female tergite VIII concave basally and truncate apically (Fig. 5f); sternite VIII rounded apically and emarginated medially, antecostal suture slightly sinuate (Fig. 5g); spermatheca with elongate pear-shaped capsule and moderately long apical invagination, stem long, sinuate and looped posteriorly, with slightly swollen apical part (Fig. 5e).

#### Distribution.

Known from Quebec.

#### Collection and habitat data.

Most adults were collected in dead black spruce logs in boreal black spruce forests, and one was captured in an intercept trap in a boreal forest during July.

### 6. Dinaraea angustula

(Gyllenhal)

http://species-id.net/wiki/Dinaraea_angustula\according to Klimaszewski et al 2013

Fig. 6a–g , Map 5

Aleochara angustula Gyllenhal, 1810: 393; as *Dinaraea*: Klimaszewski et al. 2011: 159.

#### Diagnosis.

**Figure 6.**
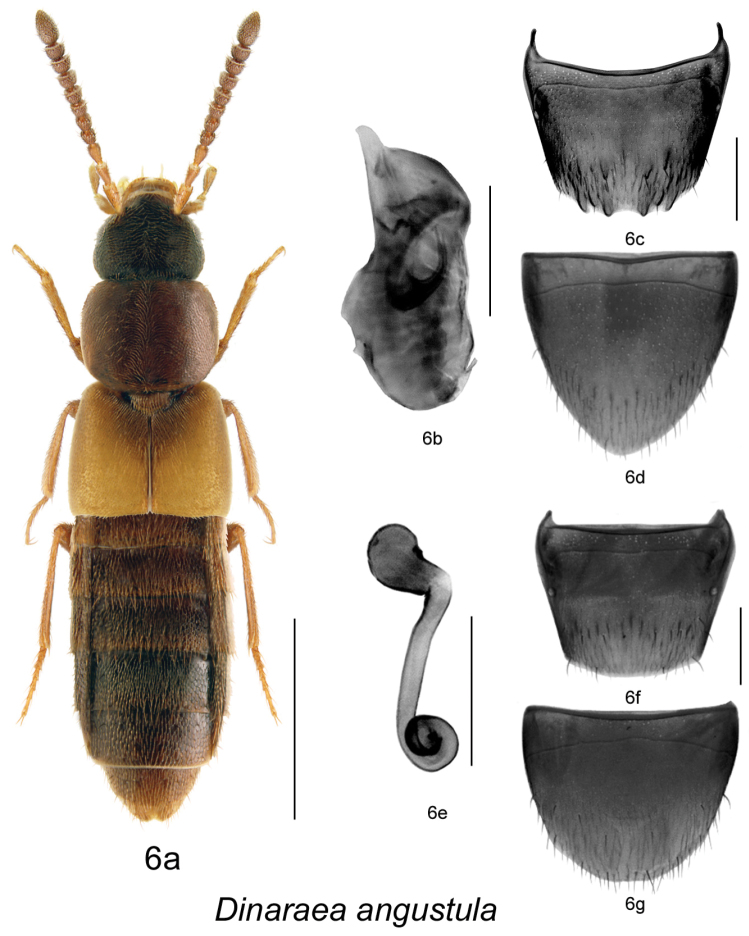
*Dinaraea angustula* (Thomson): **a** habitus **b** median lobe of aedeagus in lateral view **c** male tergite VIII **d** male sternite VIII **e** spermatheca in lateral view **f** female tergite VIII **g** female sternite VIII. Habitus scale bar = 1.0 mm, other scale bars = 0.2 mm.

*Dinaraea angustula* (habitus Fig. 6a) may be distinguished from congeners by the following combination of characters: body length 3.3–3.7 mm; head, pronotum and elytra slightly glossy with dense microsculpture; pronotum broadest at middle and narrowest at apex and base; elytra at suture as long as or slightly shorter than pronotum, with dense punctation similar to that on pronotum; antennal articles 7–10 moderately transverse; male tergite VIII with four small apical teeth, all short and rounded and some additional tooth-like subapical structures (Fig. 6c); median lobe of aedeagus with straight and short tubus narrowly rounded apically and sometimes slightly hooked at apex (Fig. 6b); spermatheca with short pear-shaped capsule, and small apical invagination, stem long, straight medially and looped posteriorly, with slightly swollen apical part (Fig. 6e).

#### Description.

Body length 3.3–3.7 mm; body dark brown with legs, antennae (at least basally), labial palpi and elytra yellowish- or reddish-brown, pronotum and abdomen lighter than head, sometimes entire body appears brown; head, pronotum and elytra slightly glossy, with dense microsculpture; abdominal microsculpture less dense and integument more glossy than that of pronotum and elytra; head about as broad as pronotum, genae slightly longer than eyes in dorsal view; pronotum broadest at middle, slightly transverse, usually longer than elytra at suture; elytra transverse, truncate posteriorly; abdomen subparallel; male tergite VIII with four small apical teeth, all short and rounded and some additional tooth-like subapical structures (Fig. 6c), sternite VIII produced apically (Fig. 6d); median lobe of aedeagus with short and straight venter of tubus and narrowly ventrally pointed apex (Fig. 6b); female tergite VIII concave basally and truncate apically (Fig. 6f), sternite VIII rounded apically and emarginated medially, antecostal suture slightly sinuate (Fig. 6g); spermatheca with short pear-shaped capsule, and small apical invagination, stem long, straight medially and looped posteriorly, with slightly swollen apical part (Fig. 6e).

#### Distribution.

This Palaearctic species is adventive in North America (Klimaszewski et al. 2010). The earliest Canadian records are: Elora, Ontario in 1975 (DEBU); St. Andrews, New Brunswick in 1978 (DEBU); Fairview, Alberta in 1982 (DEBU). The first North American records are: Buffalo and New York before 1889 (Fauvel 1889); Davis, California before 1984 (Muona 1984). In Canada, the species is known from Newfoundland and Labrador, Nova Scotia, Prince Edward Island, New Brunswick, Quebec, Ontario, Alberta and Yukon Territory (Klimaszewski et al. 2007, 2010, 2011, 2012, Majka and Klimaszewski 2008, Webster et al. 2009). In the USA, it is reported from New York, Pennsylvania and California, but is likely more widely distributed in the northeastern states.

**Map 5–6.**
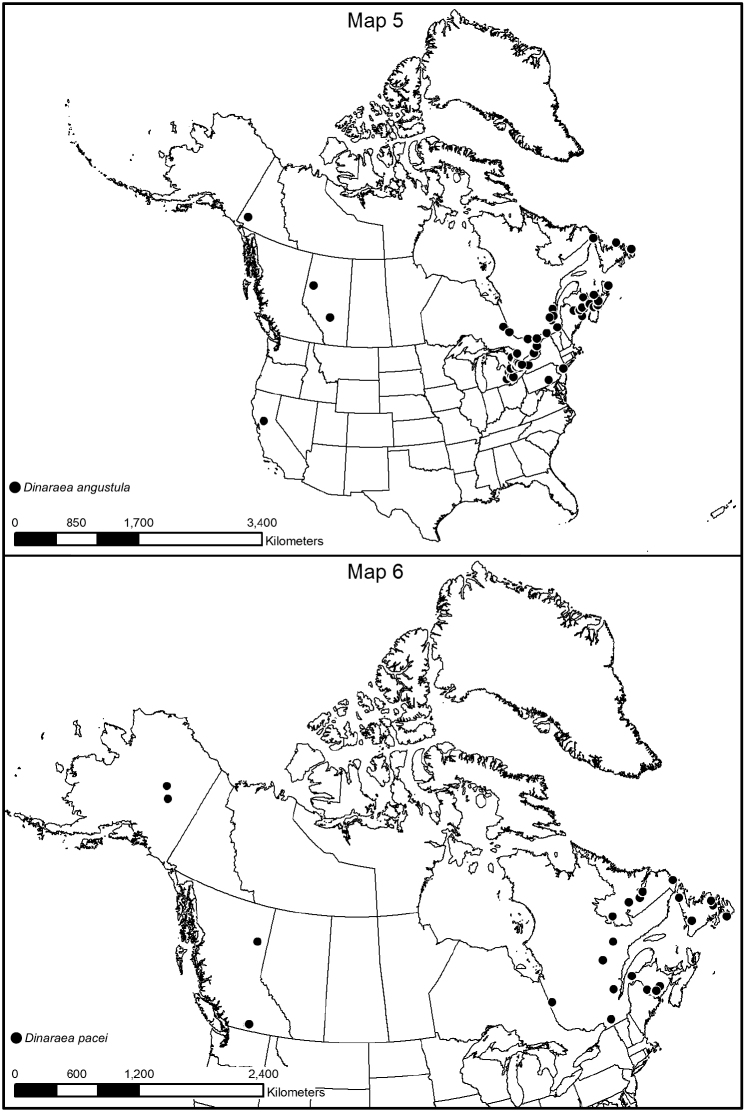
Distribution map of: **5** *Dinaraea angustula* sp. n. **6** *Dinaraea pacei*.

#### Collection and habitat data.

In North America, this species is associated with soil and organic debris in agricultural fields and disturbed urban meadows. It is also found in marsh litter, in leaf litter in mixed forests, in compost, under bark of decaying spruce logs, amongst vegetation on a coastal sand dune, in litter in a cattail marsh, in leaf litter along a vernal pond, and in drift material along a lakeshore (Webster at al. 2009, Klimaszewski et al. 2010, 2011, 2012). Illustrations and a description of the larva were published in Topp (1975).

#### Material examined.

**CANADA**, **NOVA SCOTIA**: Kings County, Sheffield Mills, July 22, 2002, Pitfall trap, Ken Neal (LFC) 1 male; Hants Co., Upper Rawdon, VII.18, 2008, J. Renkema, highbush blueberry field R2T1E (LFC) 1 male; Cape Breton Highlands National Park, Lone Shielding, PG729861, 28.VI.1983, R. Vockeroth, Malaise (CNC) 1 sex?; Bible Hill, 45.376°N, 63.260°W, 2005 (CNC) 1 sex?; Sheffield Mills, 45.151°N, 64.477°W, 1998 (NSM) 1 sex?; Sydney Tar Ponds, 46.150°N, 60.167°W, 1996 (NSM) 1 sex?; Upper Rawdon, 45.068°N, 63.712°W, 2005 (NSM) 1 sex? **NEW BRUNSWICK**: Albert Co., Shepody NWA, Germantown Section, 45.7056°N, 64.7642°W, 2004 (NBM) 1 female; Carleton Co., Wakefield (Belleville), Meduxnekeag Valley Nature Preserve, 46.1965°N, 67.6340°W, 11.V.2005, M.-A. Giguère & R. Webster // Mixed forest, margin of vernal pond in leaf litter (RWC) 1 female; Charlotte Co., St. Andrews, 45.067°N, 67.033°W, 1978 (DEBU) 1 sex?; Kent Co., N.P., Kouchibouguac, 24.V.1977, S.J. Miller, Code 51370 (CNC) 2 females; Queens Co., Canning, Grand Lake near Scotchtown, 45.8762°N, 66.1816°W, 30.IV.2006, R.P. Webster // Lake margin, in drift material (RWC) 1 female; York Co. New Maryland, Charters Settlement, 45.8395°N, 66.7391°W, 22.VII.2006, 17.IX.2006, 5.IX.2007, 23.IV.2008, 19.IX.2010, R.P. Webster // Mixed forest, in pile of decaying (mouldy) corncobs and corn husks (RWC) 2 males, 3 females. **NEWFOUNDLAND and LABRADOR**: Red Bay, 51.734°N, 56.426°W, 2009 (MUN) 1 sex?; Shalloway, 49.409°N, 53.869°W, 2001 (MUN) 1 sex?; St. John’s, Agriculture Canada Research Centre, 47.516°N, 52.785°W, 1982 (AAFC-SJ) 1 sex? **PRINCE EDWARD ISLAND**: Harrington, 46.350°N, 63.168°W, 1996 (ACPE) 1 sex? **QUEBEC**: Cookshire, Co. Compton, SAB, Lum. 1, 13-V-1996 // 96-3-0218, Downey R., Plantation (LFC) 1 sex?; same data except: Lum. 4, 23-V-1996 / 96-3-0277 (LFC) 1 sex?; Lum. 2, 3-VI-1999 / 96-3-0242 (LFC) 1 female; Lum. 5, 8-VII-1996 // 96-3-0308 (LFC) 1 sex?; 45.420°N, 71.630°W (lit) (LFC) 1 sex?; Dorval, 11-IV-1985, in deciduous forest, LeSage & Smetana (CNC) 1 male; Montreal, VII-22-1969 & VII-30-1972, E.J. Kiteley (CNC) 2 sex?; Portneuf Co., Deschambault, 30.III.2007, Coll. Michel Racine // Sous rocher et débris, talus boisé déneigé, en bordure carrière (LFC) 1 female; Réserve Portneuf, Lac Poissonneux, 47.049°N, 72.125°W, 2000 (LFC) 1 sex?; 47.050°N, 72.120°W, 2000 (LFC) 1 sex?; ZEC B-Nelson, Lac-des-Étangs, 46.963°N, 72.050°W, 2000 (LFC) 2 sex?; Co. Charlevoix, Rte. 381, 47°42'N, 70°44'W, 13–21.VI.2000, Grands-Jardins 2000 / Coupe 53 (CP53), Luminoc, piège-fosse, Pessière noire, brulée, Coupe 50 ans, 2000-3-0053 (LFC) 1 female; Québec Co., Charlesbourg, 8.X.2007, Coll. Michel Racine // Sous debris végétaux en bordure plate-bande de fleurs cultivées (LFC) 1 female; Downey River Plantation, 1996, 1 sex?, P. Downey, Lum. 1, 23–27.6.96 (LFC) 2 males. **ONTARIO**: 3 mi N. Ramore, 1–14.VIII.1973, J. Redman & C. Starr (LFC) 1 female; Hamilton, 10-13-VII.1980, M. Sanbourne (CNC) 1 female; same data except: 15.VII.1981 (CNC) 1 sex?, 19.VII.1982 (CNC) 1 sex?; Pinery Pr. Pk., Grand Bend, 16–17.V.79, M. Sanbourne (CNC) 3 sex?; Guelph, 30.VII–13.VIII.1982, D. Yu (CNC) 1 male; 18.VI.1982 (LFC) 1 male; Guelph, 43.536°N, 80.229°W, 1977 (DEBU) 1 sex?; 5 mi. Wye, VI-VII-1973, J. Redner & C. Starr (CNC, LFC) 3 males; same data except VII-VIII-1973 (CNC) 1 female; New Liskeard, 10.VII-1.VIII.1973, J. Redner & C. Starr (CNC) 1 male; Rondeau Pr. Pk., South Beach, 5.VI.1985, A. Davies, J.M. Campbell // In debris on beach at high water line (CNC) 1 male; Rondeau Pr. Park, sandy beach, 31.V.1985, A. Smetana (CNC) 4 females; Ancaster, 28.III.1963, J.E.H. Martin (CNC, LFC) 1 male, 27 sex?; Leeds Co., Chaffeys Locks, Lake Opinicon, mossy shore, 18.V.1975, I.M. Smith (CNC) 1 female; Ottawa, Kanata, 9.VII.79, A. Smetana (CNC) 1 sex?; Pr. Edward Co. (CNC) 24.X.1920, Brimley // J.F. Brimley Collection, Bequeathed 1976 (CNC) 1 male; same data except: 27.3.21 (LFC) 1 sex?; 8.V.46 (LFC) 1 sex?; Chatham, 22.V.1957, L.A. Miller (CNC, LFC) 2 males, 4 females; Windsor, Prairie area, 18.V–6.VII.1976, Dondale & Redner (CNC) 1 male; Windsor, prairie-oak woodland, 28.VI-18.V.1976, Dondale & Redner (CNC) 1 sex?; same data except: 8.VII-27.VIII.1976, pitfall (CNC) 1 female, 4 sex?; Pin oak woods (CNC) 8 sex?; Blair, Rare, The Dells, 43.383°N, 80.388°W, 2006 (DEBU) 1 sex?; Chatham, 42.434°N, 82.129°W, 2007 (DEBU) 1 sex?; Elora, 43.685°N, 80.427°W, 1975 (DEBU) 1 sex?; Owen Sound, 44.570°N, 80.930°W, 1978 (DEBU) 1 sex?; Pembroke, 45.820, 77.III.1980 (CNC) 1 sex?; Sarnia, 42.987°N, 82.318°W, 1983 (DEBU) 1 sex?; Tavistock, 43.322°N, 80.836°W, 1983 (DEBU) 1 sex?; Wilde Lake Bog, 8 km E Arthur, 43.846°N, 80.447°W, 1987 (DEBU) 1 sex?; Cottage Beaulieu, Beaulieu, 14.IV.06 (CNC) 1 sex? **ALBERTA**: Lacombe, Ag. Canada Sta., 52°28'N, 0113°44'W, 20.VII.2001, Jim Broatch // Pitfall Group EXP 53, Canada plots (harvested in 2001) VII.20-119 and VII.20-107 (LFC) 2 females; Lacombe, 54.467°N, 113.733°W, 2001 (lit) (LFC) 1 sex?; Fairview, 56.067°N, 118.384°W, 1982, (DEBU) 1 sex? **YUKON**: Alaska Hwy., Burwash Creek, 61.35°N, 139°W, 1987 (DEBU) 1 sex? **USA, PENNSYLVANIA**: Harrisburg, 22.IV.1980, E.J. Kiteley (CNC) 1 female.

### 7. Dinaraea pacei

Klimaszewski & Langor

http://species-id.net/wiki/Dinaraea_pacei

Fig. 7a–g , Map 6

Dinaraea pacei Klimaszewski & Langor, in Klimaszewski et al. 2011: 159.

#### HOLOTYPE

(male): **CANADA**, **NEWFOUNDLAND**: **LABRADOR**, Goose Bay, Rts. 500 and 520 jct., 53°16.9'N, 60°24.6'W, 13–26.VIII.2001, S. and J. Peck // flight intercept trap, elevation 10 m, spruce-poplar forest, 2001-45 (LFC). Holotype examined.

#### Diagnosis.

**Figure 7.**
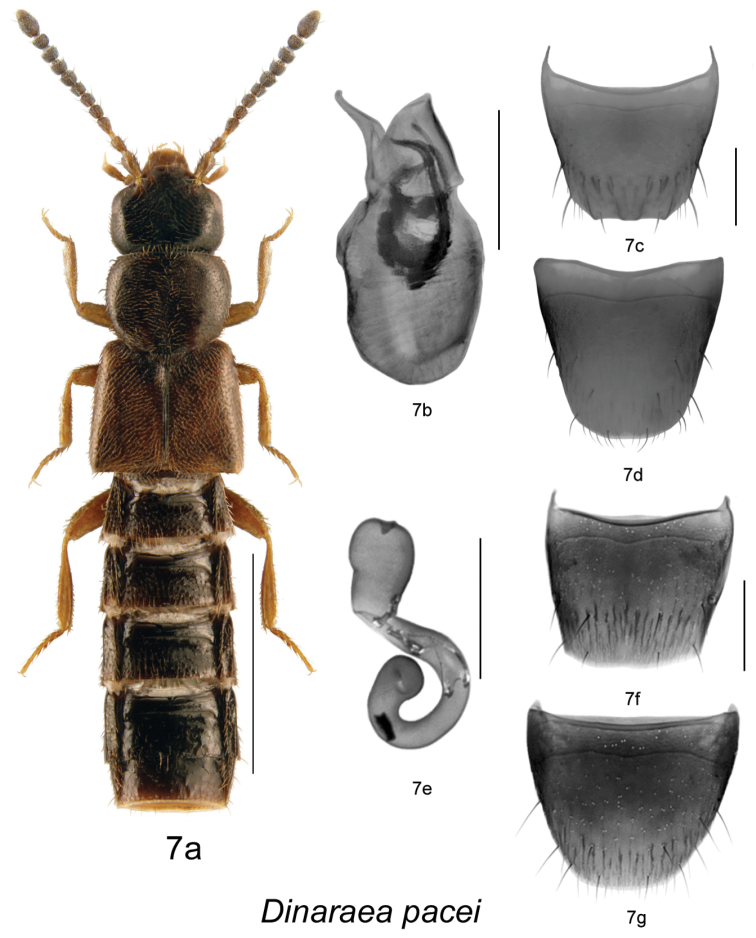
*Dinaraea pacei* Klimaszewski & Langor: **a** habitus **b** median lobe of aedeagus in lateral view **c** male tergite VIII **d** male sternite VIII **e** spermatheca in lateral view **f** female tergite VIII **g** female sternite VIII. Habitus scale bar = 1.0 mm, other scale bars = 0.2 mm.

*Dinaraea pacei* (habitus Fig. 7a) may be distinguished from congeners by the following combination of characters: body length 2.2–2.5 mm; head, pronotum and elytra slightly glossy with dense microsculpture; pronotum broadest in apical third and narrowest at base; elytra at suture slightly longer than pronotum, with dense punctation similar to that on pronotum; antennal articles 7–10 moderately transverse; male tergite VIII with four minute apical teeth, all short and rounded, and scarcely visible (Fig. 7c); median lobe of aedeagus with strongly produced ventrally and narrowly rounded apically tubus (Fig. 7b); spermatheca with short pear-shaped capsule, and small apical invagination, stem short and looped posteriorly, with slightly swollen apical part (Fig. 7e).

*Dinaraea pacei* is not distinguishable externally from *Dinaraea subdepressa* (Bernhauer), which was described from New Hampshire. However, the two species can be readily separated by the structures of the internal sac of the median lobe. The internal sac of *Dinaraea subdepressa* has two simple, straight, narrowly elongate sclerites (Fig. 13a), while the sclerites of *Dinaraea pacei* are complex as illustrated in Fig. 7b. The apical part of the median lobe is straight and slightly produced ventrally at the apex in *Dinaraea subdepressa* (Fig. 13a), while it is sinuate and strongly produced ventrally in *Dinaraea pacei* (Fig. 7b). The two species apparently represent sister taxa.

#### Description.

Body length 2.2–2.5 mm; body dark brown with legs, antennae (at least basally), labial palpi and elytra slightly paler and appearing reddish-brown, pronotum and abdomen lighter than head, sometimes entire body appears brown; head, pronotum and elytra slightly glossy, with dense microsculpture; abdominal microsculpture less dense and integument more glossy than pronotum and elytra; head about as large and broad as pronotum, genae slightly longer than eyes in dorsal view; pronotum broadest in apical third, slightly transverse, usually shorter than elytra at suture; elytra transverse, truncate posteriorly; abdomen subparallel; male tergite VIII with four minute apical teeth, all short and rounded, and scarcely visible (Fig. 7c); sternite VIII arcuate apically (Fig. 7d); median lobe of aedeagus with short and sinuate tubus and narrowly dorsally pointed apex (Fig. 7b); female tergite VIII concave basally and truncate apically (Fig. 7f), sternite VIII rounded apically and emarginated medially, antecostal suture slightly sinuate (Fig. 7g); spermatheca with short pear-shaped capsule, and small apical invagination, stem short and looped posteriorly, with slightly swollen apical part (Fig. 7e).

#### Distribution.

This species was previously recorded only from southwest Labrador (Klimaszewski et al. 2011). Here, we provide new distribution data from British Columbia, New Brunswick, Quebec and Alaska.

#### Collection and habitat data.

Adults in Newfoundland and Labrador were collected from June to August using pitfall traps and flight intercept traps in various coniferous forest types, and one specimen was collected under the bark of a dead red pine (Klimaszewski et al. 2011). In British Columbia, adults were caught in July and September in emergence traps attached to the trunks of lodgepole pine (*Pinus contorta* Dougl. ex Loud. *latifolia* Engelm.) infested by mountain pine beetle (*Dendroctonus ponderosae* Hopkins). In New Brunswick, adults were found: under the bark of large fallen spruce in an old-growth eastern white cedar swamp; under tight bark of American elm; in a silver maple forest; in fleshy polypore fungi at the base of a dead standing *Populus* sp. in a wet alder swamp; and in a group of *Pholiota* sp. at the base of a dead *Populus* sp. in a mixed forest. In Quebec, adults were found in dead black spruce in a black spruce forest. Adults were also captured in Lindgren funnel traps deployed in an old-growth white spruce (*Picea glauca* (Moench) Voss) and balsam fir forest, an old mixed forest with red and white spruce, red and white pine (*Pinus strobus* L.), and a rich Appalachian hardwood forest with some conifers. Adults were collected from March to September.

#### Material examined.

**PARATYPES**: **CANADA**, **NEWFOUNDLAND**: SW Labrador, 76 km E Churchill Falls, Rt. 500, km 345, 53°18.8'N, 62°57.9'W, 12–26.VIII.2001, S. and J. Peck // flight intercept trap, elevation 530 m, spruce-moss forest, 2001-40 (LFC) 1 male; Labrador, 75 km SW Goose Bay, Rt. 500, 53°02.6'N, 61°16.6'W, 13–26.VIII.2001, S. and J. Peck // Flight intercept trap, elevation 100 m, spruce-lichen forest, 2001-43 (LFC) 1 female; SW Labrador, 72 km E Labrador City, Rt. 500, km 93, 53°08.6'N, 66°05.9'W, 12–27.VIII.2001, S. and J. Peck // Flight intercept trap, elevation 600 m, spruce-moss forest, 2001-34 (LFC) 1 female; NW Newfoundland, Doctor’s Hill, St. John Bay, No. 185, 29.VII.1949, Ernst Palmén (MZH) 1 female; Little Grand Lake, Bakeapple Brook, old fir, pitfall, 13.VII-15.VIII.1992 (CFS-CB) 1 female; Avalon Pen., Cape St. Mary’s, 7.VI.1978, D. Larson, Lot 5, (MUN) 1 male; Grand L., 6.VI.1984, D. Langor, (MUN) 1 male; 3.5 km E. Gambo Junction, 1.VI.1982, Langor and Raske, under red pine bark, L1, (MUN) 1 female.

**NON-TYPES**: **CANADA, BRITISH COLUMBIA**: 13 km E of Princeton, 49.5056°N, 120.3097°W, ex. MPB trap on pine, col. Bleiker, 27 Sept. 2011 // MPB Predator Study, PR5-S104, September 27, 2011, Emerge @ 70° (NOFC) 1 male; 27 km NE Princeton, 49.5640°N, 120.1477°W, ex. MPB trap on pine, col. Bleiker, 28 Sept. 2011 // MPB Predator Study, PR7-S102, September 28, 2011, Emerge @ 290° (NOFC) 1 female; 82 km N Fort St. John, 56.8054°N, 121.7437°W, ex. MPB trap on pine, col. Bleiker, 13 July 2011 // MPB Predator Study, FSJ1-S103, July 13, 2011, Emerge @ 46° (NOFC) 1 female; same except FSJ1-S102, Emerge @ 150° (NOFC) 1 female. **NEW BRUNSWICK**: Carleton Co., Belleville, Meduxnekeag Valley Nature Preserve, 46.1897°N, 67.6710°W, 12.IX.2008, R.P. Webster // Mixed forest, in group of *Pholiota* sp. at base of *Populus* sp. (RWC) 1 female; Carleton Co., Jackson Falls, “Bell Forest”, 46.2200°N, 67.7231°W, 21–28.VI.2009, R. Webster & M.-A. Giguère // Rich Appalachian hardwood forest with some conifers, Lindgren funnel trap (RWC) 1 female; Queens Co., Cambridge, W of Jemseg at ``Trout Creek``, 45.8227°N, 66.1240°W, 3.VI.2007, R.P. Webster // Silver maple forest, under tight bark of *Ulmus americana* (RWC) 1 male; Restigouche Co., Dionne Brook P.N.A., 47.9064°N, 68.3431°W, 31.V–15.VI.2011, M. Roy & V. Webster // Old-growth white spruce and balsam fir forest, Lindgren funnel trap (RWC) 1 female; York Co., 8.4 km W of Tracy, off Rt. 645, 45.6821°N, 66.7894°W, 6.V.2008, R.P. Webster // Wet alder swamp in fleshy polypore fungi at base of dead standing *Populus* sp. (RWC) 1 male; York Co., 14 km WSW of Tracy, S of Rt. 645, 45.6741°N, 66.8661°W, 26.V–2.VI.2010, 2–16.VI.2010, R. Webster & C. MacKay // Old mixed forest with red and white spruce, red and white pine, balsam fir, eastern white cedar, red maple and poplar forest, Lindgren funnel traps (RWC) 2 females. **QUEBEC**: MRC Manic, Lac Lacoursière, 51.28°N, 67.99°W, 18–26.VI.2007, Chaire Côte-Nord, J.P. Légaré; Block Arbec, CP1 (CJST) 3–10 m, Multi-Pher, fosse passif, 2007-3-0396 (LFC) 1 male; Ste Julie, 2.IV.1995 (LFC) 1 female; Villebois, *Picea mariana*, J. Jacobs, 2008 (LFC) 2 males, 19 females; Lac-St-Jean, Compagnie forestière Arbec, 50°22'54"N, 70°33'29"W, 3.VI–16.VI.2009, Annelage 2009, C. Hébert, pessière à mousses (LFC) 1 female; Lac-St-Jean, Compagnie forestière Arbec, 50°21'22"N, 70°31'17"W, 4.VI–18.VI.2009, Annelage 2009, C. Hébert, pessière à mousses (LFC) 2 females; Lac-St-Jean, Compagnie forestière Arbec, 50°22'24"N, 70°33'29"W, 16.VI–01.VII.2009, Annelage 2009, C. Hébert, pessière à mousses (LFC) 5 females; Lac-St-Jean, Compagnie forestière Arbec, 50°21'22"N, 70°31'17"W, 18.VI–02.VII.2009, Annelage 2009, C. Hébert, pessière à mousses (LFC) 1 female; Lac-St-Jean, Compagnie forestière Arbec, 502°2'37"N, 703°3'03"W, 01.VII-14.VII.2009, Annelage 2009, C. Hébert, pessière à mousses (LFC) 1 female; Mare-du-Sault, Parc des Laurentides, 2700’, 15–17.VIII.1970, J.M. & B.A. Campbell (CNC) 1 male, 1 female.

**USA, ALASKA**: Fairbanks, 930 Fitz Ct., el. 280 m, 64.901296°N, 147.528609°W, +/- 15 m small stand *Betula* & *Pop. trem*., FIT, 29 IV–29 V.2008, D. Sikes, UAM100032370 (UAM) 2 females; 35.5 mi Dalton Hwy, el. 271 m, 65.74425°N, 149.34564°W, +/- 4 m *Betula*, *Salix*, white spruce, FIT, 31 V-5 VI.2008, D.S. Sikes, UAM100024452 (UAM) 1 female.

**Comments**. Several attempts, lasting about one year, to borrow lectotype of *Dinaraea planaris* (Mäklin), described from Alaska, and housed in the Museum of Helsinki have failed because the specimen is on loan and there are difficulties to get it back. This is the reason that we were not able to establish a concept of this species based on the lectotype. There is one specimen from Yukon (Dempster Hwy. mi 42, N of Klondike Riv., 1978, CNC, Ottawa), which Lohse and Smetana (1985) compared with the lectotype of *Dinaraea planaris* and according to them it is conspecific with this species. We have examined this specimen including its median lobe of aedeagus in dorsal and lateral view. Lohse and Smetana (1985) used this specimen for illustrating the median lobe of *Dinaraea planaris* in ventral view. The Yukon specimen examined by Lohse and Smetana is similar externally to *Dinaraea pacei* but differs from it by differently shaped median lobe of aedeagus (Fig. 11b). The male tergite VIII of the Yukon specimen is damaged and only about half of it is well preserved, it has small lateral tooth and minute crenulations from tooth towards the midline of disc.

### 8. Dinaraea borealis

Lohse

http://species-id.net/wiki/Dinaraea_borealis

**Figure 8.**
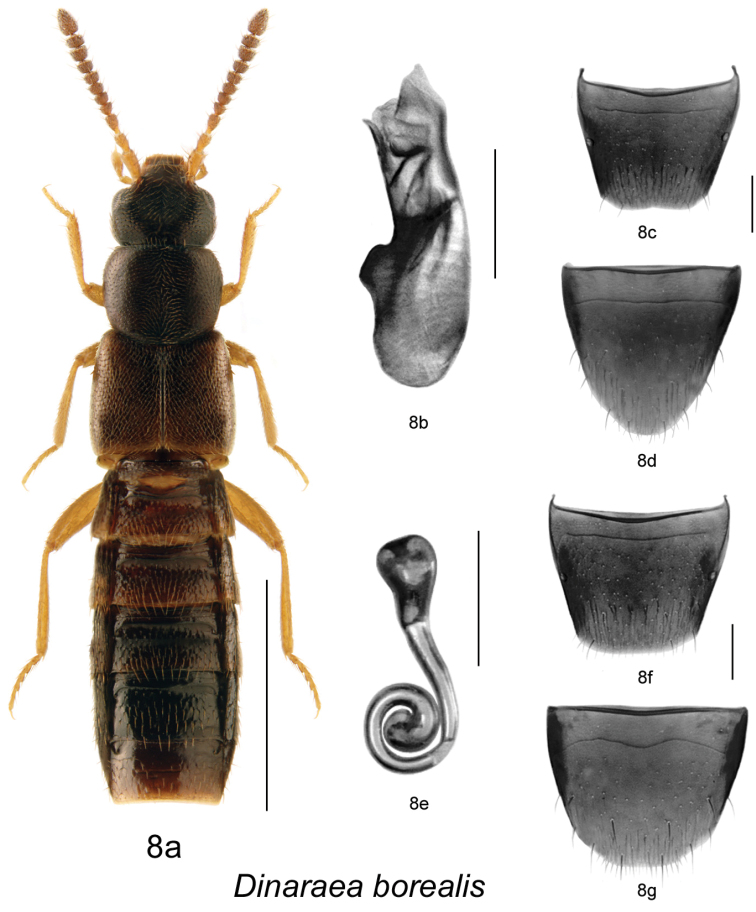
*Dinaraea borealis* Lohse: **a** habitus **b** median lobe of aedeagus in lateral view **c** male tergite VIII **d** male sternite VIII **e** spermatheca in lateral view **f** female tergite VIII **g** female sternite VIII. Habitus scale bar = 1.0 mm, other scale bars = 0.2 mm.

Fig. 8a–g , 12 , Map 8

Dinaraea borealis Lohse, in Lohse et al. 1990: 198 (based on a single female description).

#### HOLOTYPE

(female): **CANADA**, **QUEBEC**, Gt. Whale River, 30.VI.1949, J.R. Vockeroth, No. 20341 (CNC). Holotype examined.

#### Diagnosis.

*Dinaraea borealis* (habitus Fig. 8a) may be distinguished from congeners by the following combination of characters: body length 2.8–3.0 mm; head, pronotum and elytra slightly glossy with dense microsculpture; pronotum broadest in apical third and narrowest at base; elytra at suture as long as pronotum, with dense punctation similar to that on pronotum; antennal articles 7–10 moderately transverse; male tergite VIII without apical teeth (Fig. 8c); median lobe of aedeagus with straight, long tubus pointed at apex and slightly pointed ventrally (Fig. 8b); spermatheca elongate with pear-shaped capsule, and long apical invagination, stem long and looped posteriorly, with strongly swollen apical part (Fig. 8e).

#### Description.

Body length 2.8–3.0 mm; body variable in colour, either entirely black with brown or reddish appendages and part of elytra, or dark brown with legs, antennae (at least basally), and labial palpi appearing reddish-brown; head, pronotum and elytra slightly glossy, the latter more so, with dense microsculpture; abdominal microsculpture less dense and integument more glossy than that of pronotum and elytra; head moderately large, as broad as pronotum, genae slightly longer than eyes in dorsal view; pronotum broadest in apical third, slightly transverse, usually as long as elytra at suture; elytra transverse, truncate posteriorly; abdomen arcuate laterally. MALE (**new description**): tergite VIII truncate apically and without apical teeth (Fig. 8c); sternite VIII rounded apically (Fig. 8d); median lobe of aedeagus with long and straight tubus with apex pointed ventrally (Fig. 8b). FEMALE: tergite VIII concave basally and truncate apically (Fig. 8f); sternite VIII rounded apically, antecostal suture strongly sinuate (Fig. 8g); spermatheca elongate with pear-shaped capsule, and long apical invagination, stem long and looped posteriorly, with strongly swollen apical part (Figs 8e, 12).

#### Distribution.

This species was previously recorded only from Gt. Whale River in Quebec (Lohse et al. 1990). Here, we provide new distribution data for New Brunswick, Quebec, and Ontario.

**Map 7–10.**
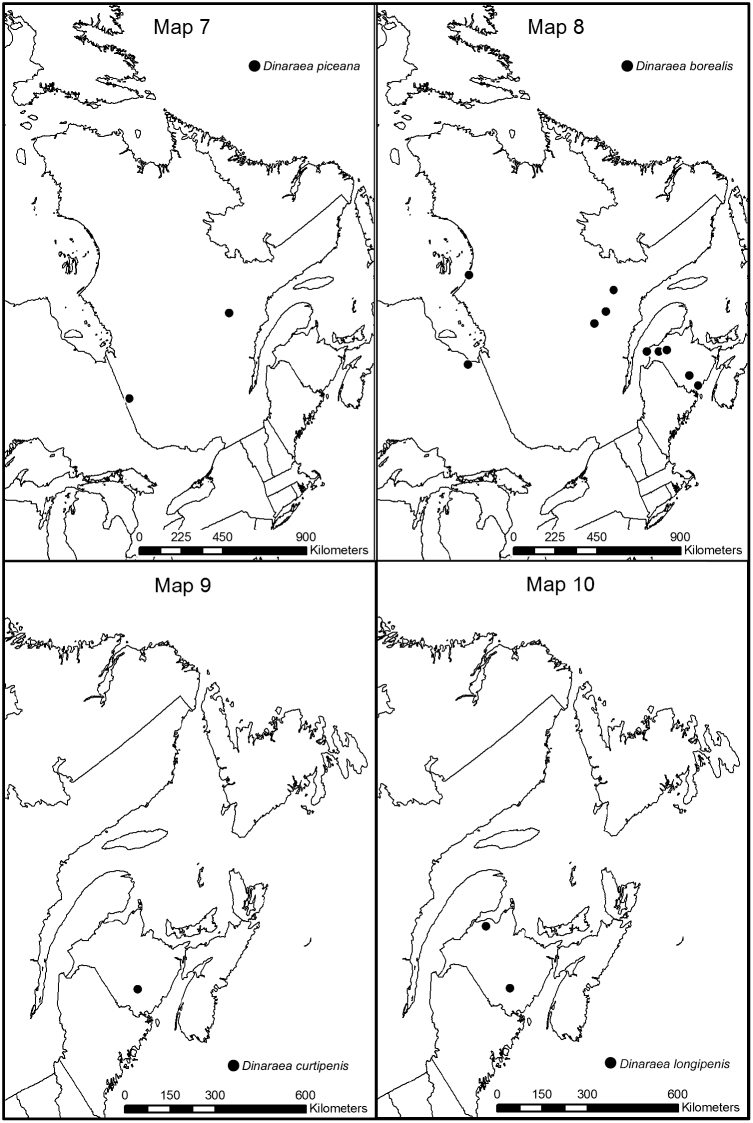
Distribution map of: **7** *Dinaraea piceana* sp. n. **8** *Dinaraea borealis* **9** *Dinaraea curtipenis* sp. n. **10** *Dinaraea longipenis* sp. n.

#### Collection and habitat data.

Adults were collected from March to August from: under bark of large fallen spruce in old-growth eastern white cedar forests; leaf litter under alders near a small stream in a mixed forest; and moss in a black spruce forest. Specimens were also captured in Lindgren funnel traps in an old-growth eastern white cedar forest and an old-growth white spruce and balsam fir forest.

#### Material examined.

**CANADA**, **NEW BRUNSWICK**: Charlotte Co., 10 km NW of New River Beach, 45.2110°N, 66.6170°W, 15–29.VI.2010, R. Webster & C. MacKay // Old-growth eastern white cedar forest, Lindgren funnel trap (LFC) 1 female; Restigouche Co., Little Tobique River near Red Brook, 47.44616°N, 67.06888°W, 24.V.2007, R.P. Webster // Old-growth eastern white cedar forest, under bark of large fallen spruce (LFC, RWC) 2 males, 2 females; Restigouche Co., MacFarlane Brook Protected Area, 47.6018°N, 67.6263°W, 25.V.2007, R.P. Webster // Old-growth eastern white cedar swamp, under bark of large fallen spruce (RWC) 1 male, 2 females; Restigouche Co., Dionne Brook P.N.A., 47.9064°N, 68.3441°W, 31.V–15.VI.2011, M. Roy & V. Webster // Old-growth white spruce and balsam fir forest, Lindgren funnel trap (RWC) 3 males, 2 females; York Co. New Maryland, Charters Settlement, 45.8395°N, 66.7391°W, 29.III.2006, R.P. Webster // Mixed forest, under alders near small stream, in leaf litter (RWC) 1 female. **QUEBEC**: MRC Manic, Réservoir Outardes 4, 50.60°N, 69.37°W, 30.VII–07.VIII.2007, Chaire Côte-Nord, J.P. Légaré, Block Abitibi Nord, Témoin 1–10 m, Piège à impact, 2007-3-1306 (LFC) 1 male; MRC Manic, Lac Lacoursière, 51.27°N, 67.99°W, 18.VI–26.VI.2007, Chaire Côte-Nord, J.P. Légaré, Bloc Arbec, Témoin 3–10 m, Piège à impact, 2007-3-0168, CPRS 4–15 m, 2007-3-01153 (LFC) 2 females; same data except: 30.VII-07.VIII.2007, CPRS 3–10 m, 2007-3-1352 (LFC) 1 female; Lac-St-Jean, Compagnie forestière Arbec, 50°22'54"N, 70°33'29"W, 3.VI–16.VI.2009, Annelage 2009, C. Hébert, pessière à mousses (LFC) 1 female; Lac-St-Jean, Compagnie forestière Arbec, 50°21'22"N, 70°31'17"W, 4.VI–18.VI.2009, Annelage 2009, C. Hébert, pessière à mousses (LFC) 2 males; Lac-St-Jean, Compagnie forestière Arbec, 50°22'54"N, 70°33'29"W, 16.VI–01.VII.2009, Annelage 2009, C. Hébert, pessière à mousses (LFC) 1 male, 2 females; Compagnie forestière Arbec, 50°22'37"N, 70°33'08"W, 17.VI–01.VII.2009, Annelage 2009, C. Hébert, pessière à mousses (LFC) 3 males, 2 females; Lac-St-Jean, Compagnie forestière Arbec, 50°21'22"N, 70°31'17"W, 18.VI–02.VII.2009, Annelage 2009, C. Hébert, pessière à mousses (LFC) 1 female. **ONTARIO**: Moosonee, 2.VII.1973, Parry & Campbell (CNC) 1 female.

### 9. Dinaraea curtipenis

Klimaszewski & Webster sp. n.

http://zoobank.org/214D0779-559A-4512-AC42-1D0B9B353A1B

http://species-id.net/wiki/Dinaraea_curtipenis

Fig. 9a–d , Map 9

#### HOLOTYPE

(male): **CANADA**, **NEW BRUNSWICK:** York Co., New Maryland, Charters Settlement, 45.8395°N, 66.7391°W, 1.V.2004, R.P. Webster // Mixed forest, under bark of conifer log (LFC). **PARATYPE: CANADA**, **NEW BRUNSWICK:** York Co., New Maryland, Charters Settlement, 45.8395°N, 66.7391°W, 22.VII.2005, R.P. Webster // Mixed forest, in well-rotted fungus-covered log (RWC) 1 male.

#### Etymology.

*Curtipenis* means ‘possessing a short penis’, in allusion to the short tubus of the median lobe of the aedeagus in this species.

#### Diagnosis.

**Figure 9.**
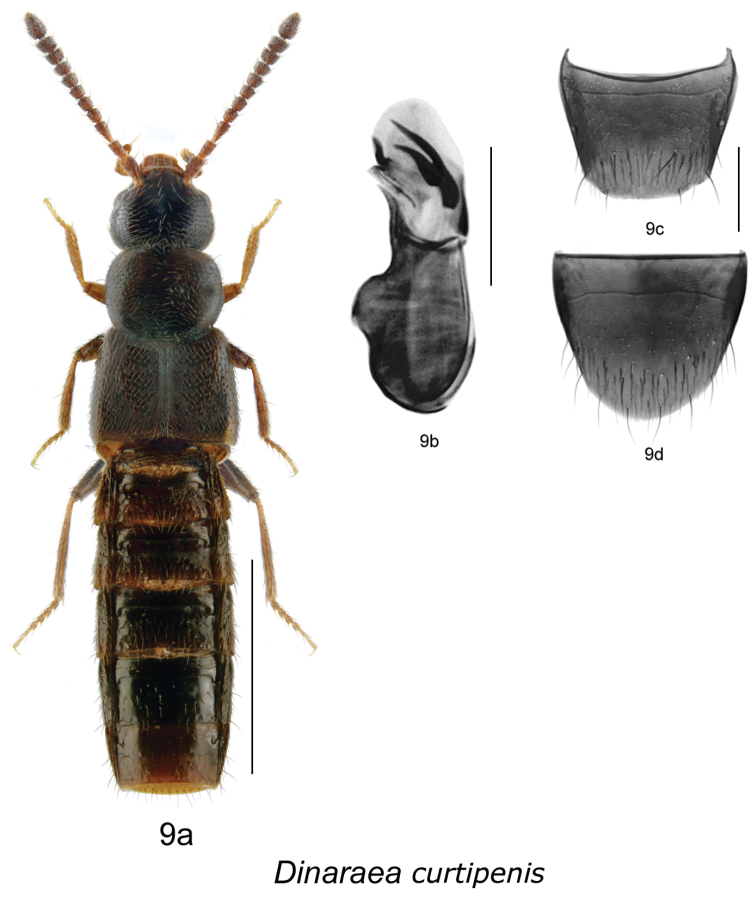
*Dinaraea curtipenis* Klimaszewski & Webster, sp. n.: **a** habitus **b** median lobe of aedeagus in lateral view **c** male tergite VIII **d** male sternite VIII. Habitus scale bar = 1.0 mm, other scale bars = 0.2 mm.

*Dinaraea curtipenis* (habitus Fig. 9a) may be distinguished from congeners by the following combination of characters: body length 3.0 mm; head, pronotum and elytra slightly glossy with dense microsculpture and bluish tinge; pronotum broadest in the middle and narrowest at base; elytra at suture as long as pronotum, with dense punctation similar to that on pronotum; antennal articles 7–10 moderately transverse; male tergite VIII without apical teeth (Fig. 9c); median lobe of aedeagus with straight, short tubus pointed at apex and slightly pointed ventrally (Fig. 9d), internal sac with distinct sclerites (Fig. 9b). *Dinaraea curtipenis* is superficially similar to *D. subdipressa* but can be distinguished from it by lacking a strongly depressed posterior edge of pronotum, while it is strongly depressed at the posterior angle of the disc and medially at base forming a groove in the latter species; and it has more elongate bulbus of median lobe of aedeagus with additional dorsal and ventral sclerotized structures (Fig. 9b).

#### Description.

Body length 3.0 mm; body dark brown with reddish-brown tarsi, tibia and bases of antennae; head, pronotum and elytra slightly glossy, elytra more so, with dense microsculpture; abdominal microsculpture less dense and integument more glossy than that of pronotum and elytra; head large, as broad as pronotum, genae slightly longer than eyes in dorsal view; pronotum broadest in middle, slightly transverse, usually as long as elytra at suture; elytra transverse, truncate posteriorly; abdomen subparallel; male tergite VIII truncate apically and without apical teeth (Fig. 9c); sternite VIII rounded apically (Fig. 9d); median lobe of aedeagus with short and straight tubus with apex pointed ventrally (Fig. 9b). Female undescribed.

FEMALE. We have several females from New Brunswick localities found without male association that may belong to this species but because they are very similar externally and have similar genitalia to those of *Dinaraea pacei* we hesitate to formally associate them with *Dinaraea curtipenis*. These females have a more coarsely punctate pronotum than those of *Dinaraea pacei* but have similar rounded posterior angles of pronotum. We anticipate that this problem will be solved by finding females associated with males at the same locality.

#### Distribution.

This species is known only from New Brunswick.

#### Collection and habitat data.

The holotype was collected from under the bark of a conifer log in a mixed forest. Other individuals were captured in Lindgren funnel traps in an old red oak forest, an old-growth eastern white cedar forest, an old-growth white spruce and balsam fir forest, and old red pine forests. The paratype was collected from a well-decayed and fungus-covered log in a mixed forest. Adults were collected during May and July.

### 10. Dinaraea longipenis

Klimaszewski & Webster sp. n.

http://zoobank.org/BE14DE6E-C25E-4DDC-8E43-60E72F3F7D69

http://species-id.net/wiki/Dinaraea_longipenis

Fig. 10a–d , Map 10

#### HOLOTYPE

(male): **CANADA**, **NEW BRUNSWICK:** York Co., New Maryland, Charters Settlement, 45.8267°N, 66.7343°W, 3.V.2006, R.P. Webster // *Carex* marsh, treading, sp. # 444 (LFC). **PARATYPE**: **CANADA**, **NEW BRUNSWICK:** Restigouche Co., Jacquet River Gorge P.N.A., 47.7491°N, 66.1114°W, 24.VI.2008, R.P. Webster // Hardwood forest, in well-rotted log (RWC) 1 male.

#### Etymology.

*Longipenis* means ‘possessing a long penis’, in allusion to the long tubus of the median lobe of the aedeagus in this species.

#### Diagnosis.

**Figure 10.**
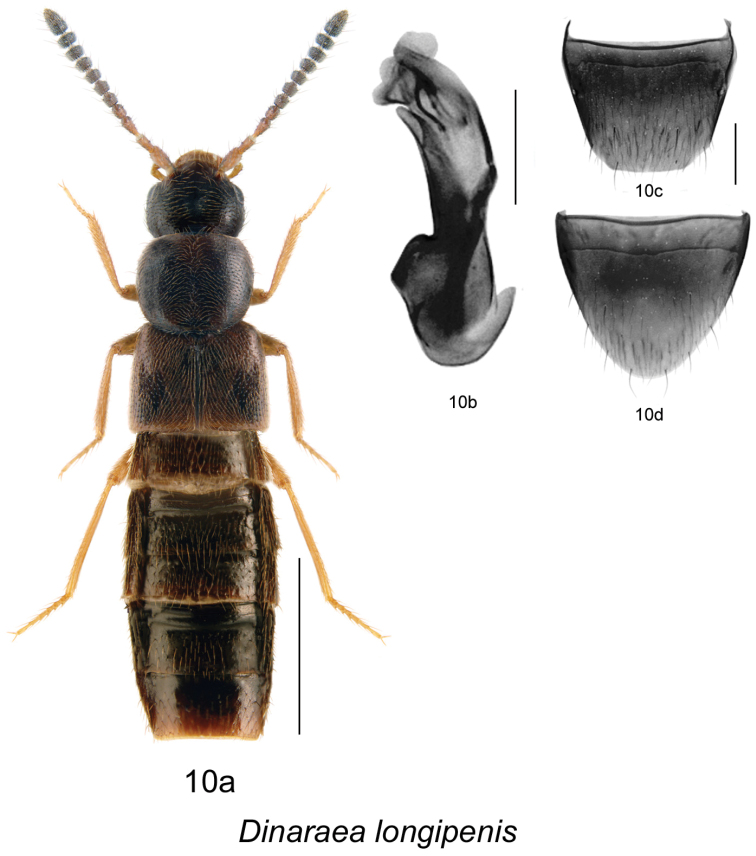
*Dinaraea longipenis* Klimaszewski & Webster, sp. n.: **a** habitus **b** median lobe of aedeagus in lateral view **c** male tergite VIII **d** male sternite VIII. Habitus scale bar = 1.0 mm, other scale bars = 0.2 mm.

*Dinaraea longipenis* (habitus Fig. 10a) may be distinguished from congeners by the following combination of characters: body length 3.5 mm; head, pronotum and elytra slightly glossy with dense microsculpture; pronotum broadest in the middle and narrowest at base; elytra at suture shorter than pronotum, with dense punctation similar to that on pronotum; antennal articles 7–10 strongly transverse; male tergite VIII without apical teeth (Fig. 10c); median lobe of aedeagus with straight, long tubus rounded at apex and slightly pointed ventrally (Fig. 10b), internal sac with thin sclerites (Fig. 10b). FEMALE: We have one female from New Brunswick found without male association that may belong to this species. This female is externally very similar to the male of *Dinaraea longipenis* but the spermatheca and tergite and sternite VIII are indistinguishable from that of *Dinaraea piceana*. We need more specimens of this species, with male and female association, to confirm and formally describe the female of this species.

**Figures 11–13.**
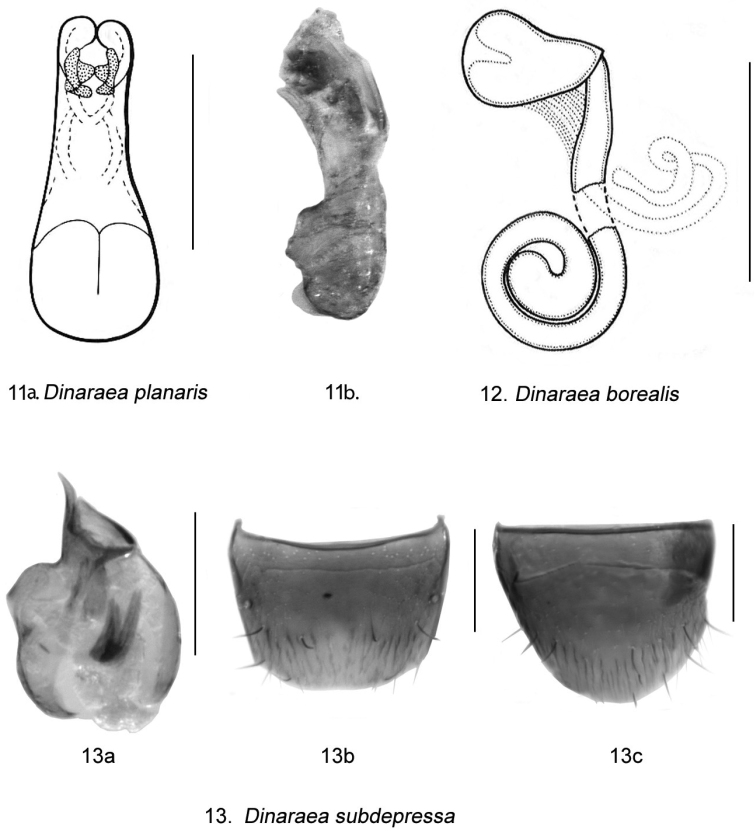
Genital structures of *Dinaraea*: **11a** *Dinaraea planaris* (Mäklin), median lobe of aedeagus in ventral view, after Lohse and Smetana (1985), and **11b** in lateral view **12** *Dinaraea borealis* Lohse, spermatheca in lateral view, after Lohse (in Lohse et al. 1990) **13a–c** *Dinaraea subdepressa* (Bernhauer), original images based on the type specimen: **13a** median lobe of aedeagus in lateral view **13b** male tergite VIII **13c** male sternite VIII. Scale bars = 0.2 mm.

#### Description.

Body length 3.5 mm; body dark black with light reddish-brown tarsi, tibia and bases of antennae; head, pronotum and elytra slightly glossy, the elytra more so, with dense microsculpture; abdominal microsculpture less dense and integument more glossy than that of pronotum and elytra; head large, about as broad as pronotum, genae slightly longer than eyes in dorsal view; pronotum broadest in middle, slightly transverse, longer than elytra at suture; elytra transverse, truncate posteriorly; abdomen arcuate laterally; male tergite VIII truncate apically and without apical teeth, slightly angular laterally at apical margin (Fig. 10c), sternite VIII rounded apically (Fig. 10d); median lobe of aedeagus with long and straight tubus with apex rounded and pointed ventrally (Fig. 10b). Female undescribed (see above).

#### Distribution.

This species is known only from New Brunswick.

#### Collection and habitat data.

The holotype was captured during May in a *Carex* marsh by treading emergent vegetation. The paratype was collected from a well-rotted log in a hardwood forest.

## Supplementary Material

XML Treatment for Dinaraea

XML Treatment for Dinaraea bicornis

XML Treatment for Dinaraea quadricornis

XML Treatment for Dinaraea backusensis

XML Treatment for Dinaraea worki

XML Treatment for Dinaraea piceana

XML Treatment for Dinaraea angustula

XML Treatment for Dinaraea pacei

XML Treatment for Dinaraea borealis

XML Treatment for Dinaraea curtipenis

XML Treatment for Dinaraea longipenis
